# Combination of Bempegaldesleukin and Anti-CTLA-4 Prevents Metastatic Dissemination After Primary Resection or Radiotherapy in a Preclinical Model of Non-Small Cell Lung Cancer

**DOI:** 10.3389/fonc.2021.645352

**Published:** 2021-04-15

**Authors:** Amber M. Bates, Ryan J. Brown, Alexander A. Pieper, Luke M. Zangl, Ian Arthur, Peter M. Carlson, Trang Le, Gustavo A. Sosa, Paul A. Clark, Raghava N. Sriramaneni, KyungMann Kim, Ravi B. Patel, Zachary S. Morris

**Affiliations:** ^1^ Department of Human Oncology, University of Wisconsin School of Medicine and Public Health, University of Wisconsin-Madison, Madison, WI, United States; ^2^ Department of Biostatistics and Medical Informatics, University of Wisconsin School of Medicine and Public Health, University of Wisconsin-Madison, Madison, WI, United States; ^3^ Departments of Radiation Oncology and Bioengineering, University of Pittsburgh Hillman Cancer Center, Pittsburgh, PA, United States

**Keywords:** NSCLC, metastasis, radiation, bempegaldesleukin, immunotherapy, IL2

## Abstract

Surgical resection or hypo-fractionated radiation therapy (RT) in early-stage non-small cell lung cancer (NSCLC) achieves local tumor control, but metastatic relapse remains a challenge. We hypothesized that immunotherapy with anti-CTLA-4 and bempegaldesleukin (BEMPEG; NKTR-214), a CD122-preferential IL2 pathway agonist, after primary tumor RT or resection would reduce metastases in a syngeneic murine NSCLC model. Mice bearing Lewis Lung Carcinoma (LLC) tumors were treated with combinations of BEMPEG, anti-CTLA-4, and primary tumor treatment (surgical resection or RT). Primary tumor size, mouse survival, and metastatic disease at the time of death were assessed. Flow cytometry, qRT-PCR, and cytokine analyses were performed on tumor specimens. All mice treated with RT or surgical resection of primary tumor alone succumbed to metastatic disease, and all mice treated with BEMPEG and/or anti-CTLA-4 succumbed to primary tumor local progression. The combination of primary tumor RT or resection and BEMPEG and anti-CTLA-4 reduced spontaneous metastasis and improved survival without any noted toxicity. Flow cytometric immunoprofiling of primary tumors revealed increased CD8 T and NK cells and decreased T-regulatory cells with the combination of BEMPEG, anti-CTLA-4, and RT compared to RT alone. Increased expression of genes associated with tumor cell immune susceptibility, immune cell recruitment, and cytotoxic T lymphocyte activation were observed in tumors of mice treated with BEMPEG, anti-CTLA-4, and RT. The combination of BEMPEG and anti-CTLA-4 with primary tumor RT or resection enabled effective control of local and metastatic disease in a preclinical murine NSCLC model. This therapeutic combination has important translational potential for patients with early-stage NSCLC and other cancers.

## Introduction

Improvements in early detection (1–4) as well as advancements in surgery and radiation therapy (RT) have led to primary tumor control rates > 90% in early-stage non-small cell lung cancer (NSCLC) (5–10). Despite these improvements, the 5-year survival for patients with localized NSCLC remains below 60% (11) because many patients achieving primary tumor control nevertheless experience regional or metastatic recurrence of disease (12, 13). Treatment approaches that effectively control clinically occult metastatic disease are therefore needed in combination with primary tumor treatments for early-stage NSCLC.

Immunotherapies that activate a patient’s own immune system to attack cancer cells have shown efficacy in the treatment of metastatic and regionally advanced NSCLC (14–18). Immune checkpoint inhibitors (ICI) are a class of monoclonal antibodies that modulate tumor tolerance among immune cells by blocking specific inhibitory receptor-ligand interactions to overcome immune exhaustion (e.g. anti-CTLA-4, anti-PD-1). In a clinical study combining hypofractionated palliative RT with ipilimumab (anti-CTLA-4) to treat patients with metastatic NSCLC, objective responses were observed in 18% of enrolled patients and 31% had disease control, but only 2 out of 39 patients had a complete response (19). Additional immunotherapy combinations that aid in preventing metastases should thus be further investigated. One promising immunotherapy is recombinant interleukin-2 (IL2) which expands antigen-specific CD8 T cell populations (20), and it has been shown to induce durable disease control in some patients with metastatic melanoma and renal cell carcinoma (21–23). However, clinical use of high-dose IL2 is limited due to its toxicity and short half-life (22, 24).

Bempegaldesleukin (BEMPEG; NKTR-214) is an investigational CD122-preferential IL2 pathway agonist that leverages the IL2 pathway to stimulate an antitumor immune response. BEMPEG, an IL2 protein with multiple releasable covalently attached polyethylene glycol (PEG) chains, is inactive upon administration, and overcomes the limitations of recombinant IL2 by providing active IL2 conjugate species *in vivo* as PEG chains are progressively released to achieve a sustained concentration of active drug and stable activity (24, 25). This drug delivery mechanism enables an improved safety profile, longer half-life, and outpatient dosing. IL2 stimulates proliferation of CD8 T cell and NK cells through the binding of the intermediate affinity IL2βγ receptor, but it also interacts with the high affinity trimeric IL2αβγ receptor leading to the expansion of regulatory T cells (Tregs), which can be immunosuppressive in the tumor microenvironment (TME) (20, 26). In patients with NSCLC, higher levels of Tregs in the TME are associated with a higher risk of recurrence (27, 28). Compared with native IL2, BEMPEG preferentially binds to the intermediate affinity IL2βγ receptor (CD122) and favors expansion for CD8 and NK cells without expansion of unwanted intratumoral Tregs (24, 29, 30).

In this preclinical study, we utilize a spontaneously metastasizing, immunologically “cold” Lewis lung carcinoma model (LLC) to test the capacity of anti-CTLA-4 and BEMPEG to prevent distant metastases after primary treatment with hypofractionated RT or surgical resection. We report a cooperative interaction between this combination of systemic immunotherapies and current standard local therapies employed against early-stage NSCLC and demonstrate the capacity of this combined treatment approach to elicit durable local and metastatic tumor control.

## Materials and Methods

### Murine Tumor Models

Wild-type female C57BL/6 mice aged 6-8 weeks were obtained from Taconic Biosciences (Germantown, NY). Mice were housed and treated in accordance with the Guide for Care and Use of Laboratory Animals under a protocol approved by our Institutional Animal Care and Use Committee. All *in vivo* experiments were duplicated to demonstrate reproducible results.

Lewis Lung Carcinoma (LLC) cells were used in all experiments and were obtained from American Type Culture Collection (ATCC^®^ CRL-1642). Cells were cultured in Dulbecco’s Modified Eagle Medium with 100 U/mL penicillin-streptomycin and 10% fetal bovine serum (FBS, ThermoFisher). Cells were transferred to new flasks when they reached 80% confluency. Early passages after thaw (3-8) were used for all experiments. Before implantation, cells were washed and resuspended with PBS to remove all media, FBS, penicillin-streptomycin, and trypsin.

**Figure 1:**
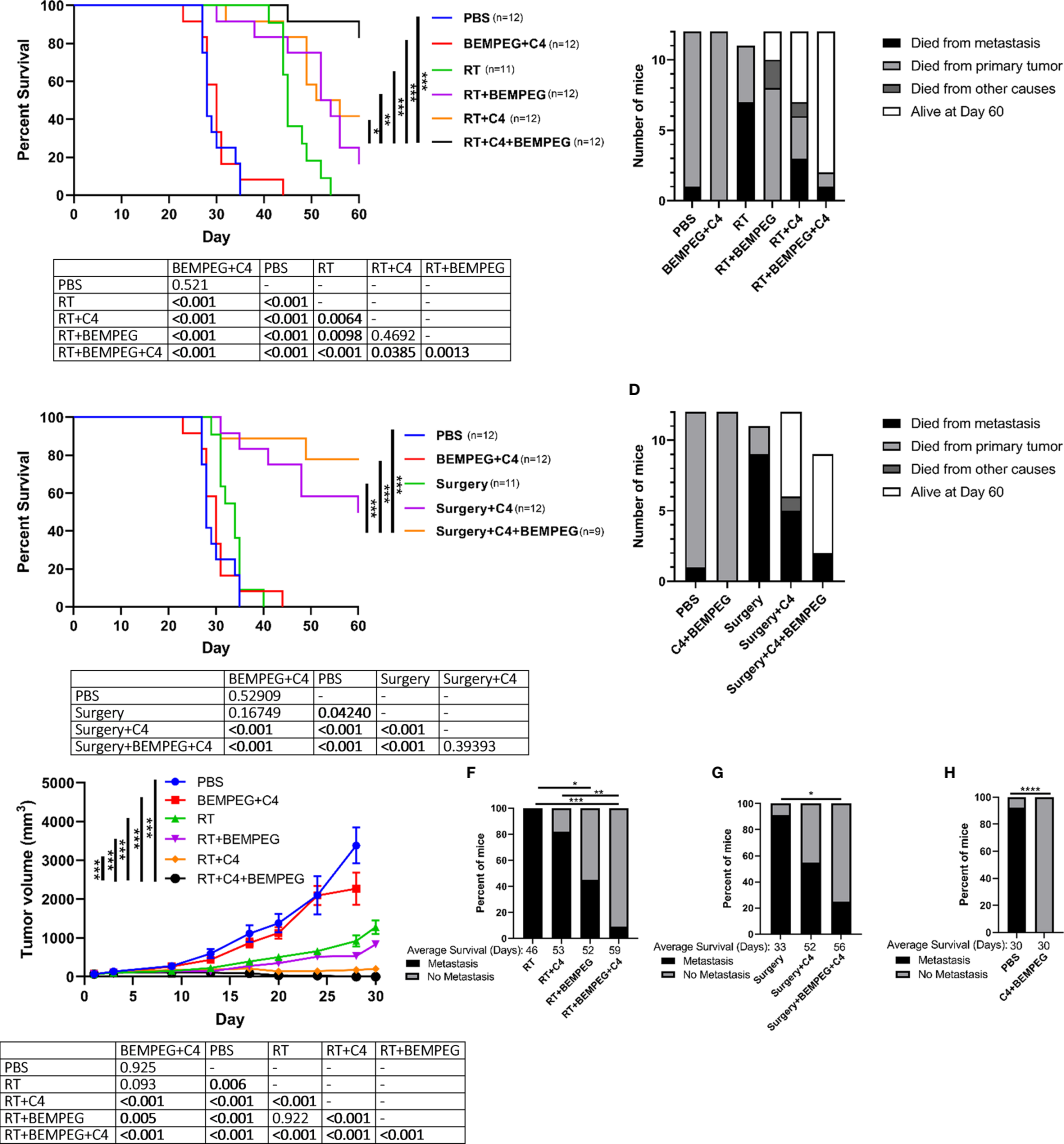
BEMPEG and anti-CTLA-4 with local tumor treatment leads to tumor regression and increased survival in a single flank tumor model. In mice bearing a flank LLC tumor (~80 mm^3^ on day 1), tumors were treated with 8 Gy x 3 daily fractions (days 1, 2, 3) or were surgically resected (day 16). Local treatment was combined with BEMPEG, anti-CTLA-4 (C4), or PBS control treatments. **(A, C)** Survival curves (Kaplan–Meier and Log-rank pairwise comparison with Benjamini-Hochberg adjustment for p-values, n≥10, 2 independent animal experiments) are shown comparing RT+C4+BEMPEG or surgery+BEMPEG+C4 to controls. **(B, D)** Cause of death is graphed for mice receiving the indicated treatments. **(E)** Tumor volume growth curves are shown comparing RT+C4+BEMPEG to controls (linear mixed effects model, mean ± SEM, n≥5, replicate experiment and individual mouse growth curves are shown in **Supplemental Figure 6**). **(F–H)** At the time of death, metastatic disease was determined *via* India ink staining of the lungs. Fisher’s exact test and a post-hoc pairwise comparison with Benjamini-Hochberg adjustment for p-values were used for statistical analyses. **** = *P* < 0.0001; *** = *P* < 0.001; ** = *P* < 0.01; * = *P* < 0.05.

**Figure 2:**
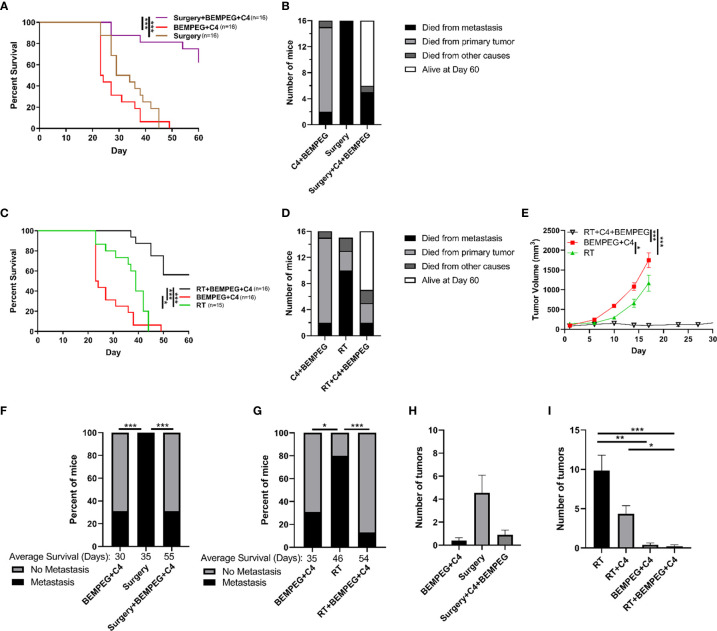
BEMPEG and anti-CTLA-4 reduce metastatic spread in a disseminated LLC model. Mice bearing a LLC primary flank tumor (80 mm^3^ on day 1) received an IV injection of 2x10^5^ LLC cells on day 10 after treatment initiation with 8 Gy x 3 daily fractions (days 1, 2, 3) or surgical resection (day 16). This local treatment was combined with BEMPEG and anti-CTLA-4 (C4) treatments. **(A, C)** Survival curves (Kaplan–Meier and Log-rank pairwise comparison with Benjamini-Hochberg adjustment for p-values, n≥9, 2 independent animal experiments) are shown. **(B, D)** Cause of death is graphed for mice receiving the indicated treatments. **(E)** Tumor volume growth curves are shown (linear mixed effects model, mean ± SEM, n=10, replicate experiment and individual mouse growth curves are shown in **Supplemental Figure 7**). **(F, G)** At the time of death, metastatic disease was determined *via* India ink staining of the lungs. Fisher’s exact test and a post-hoc pairwise comparison with Benjamini-Hochberg adjustment for p-values were used for statistical analyses. At day 22 **(H)** and day 35 **(I)** mice were euthanized (replicate experiments in **Supplemental Figure 8**), lungs were harvested, and India ink stained as shown in **Supplemental Figure 1A**, and lung metastatic tumor burden was quantified. *** = P < 0.001; ** = P < 0.01; * = P < 0.05.

Tumors were generated from intradermal injections on the dorsal right flank of the mice with 1 x 10^6^ LLC cells. Prior to each *in vivo* experiment, mice were randomized into their respective treatment. This was performed when the mean flank tumor size for the entire cohort reached ~80 mm^3^. Tumor volume was approximated as (width^2^ x length)/2 and measured biweekly using digital calipers. In the disseminated metastasis LLC model, lung metastasis was established by a tail veil injection of 2 x 10^5^ LLC cells on day 10. Mice used for immunophenotyping experiments and lung metastasis quantification were euthanized at predetermined time points. For the survival experiments in **Figures 1** and **2**, cause of death graphs by day 60 are shown in **Figures 1** and **2** to depict how the mice in the survival experiments died, whether it was from metastatic disease in which the mouse was euthanized or found dead with lung tumors (died from metastasis), euthanized due to the primary tumor size reaching 20 mm in any dimension (died from primary tumor), or if the mouse was euthanized by vet staff request due to moribund behavior or found dead with no lungs tumors (died from other causes).

After 90 days, mice from either model with a complete tumor response were rechallenged to evaluate for immune memory by an additional injection of 1 x 10^6^ LLC cells intradermally in the opposite (left) flank. Age-matched naive mice were injected in the left flank with the same number of tumor cells.

### Lung Staining

In order to visualize the lung metastasis, India ink staining was performed as previously described (31, 32). Blue India ink diluted 1:10 was injected intratracheally until the lungs were fully saturated with stain. Lungs were de-stained in Fekete’s solution (580 mL 95% ethanol, 200 mL H_2_O, 80 mL 37% formaldehyde, 40 mL glacial acidic acid) for 5 minutes after harvesting and then placed in 10% formalin to fix the tissue. After 48 hours, the stained lungs were transferred to 70% ethanol until quantification. Lung metastasis were determined by the absence of staining at tumor locations under a dissection microscope. The entire surface of each lobe was examined thoroughly from multiple angles by two independent investigators who analyzed the lungs and were blinded to the treatment conditions. Lung metastases do not stain, while healthy lung tissue stains blue, allowing lung metastases to be grossly detectable and easily quantified (**Supplemental Figure 1A**). H&E-stained sections of these representative lungs revealed histological differences in the tissue indicative of tumor (**Supplemental Figure 1B**).

### Treatments

Tumor targeted external beam RT was delivered using an XRad320 (PXi) irradiator (Precision X-Ray, Inc., North Branford, CT) in three daily fractions of 8 Gy on what was defined as treatment days 1, 2, and 3. Mice were immobilized and normal tissues outside of the right dorsal flank were shielded during RT using custom lead blocks. Surgical removal of tumors occurred on day 16. Mice were anesthetized *via* isoflurane and wounds were closed using staples. Anti-mouse-CTLA-4 mAb (IgG2c isotype of the 9D9 clone), provided by Bristol-Myers Squibb (Redwood City, CA), was administered *via* an intraperitoneal injection of 200 μg on treatment days 4, 7, and 10. Bempegaldesleukin (BEMPEG; NKTR-214), provided by Nektar Therapeutics (San Francisco, CA), was administered intravenously by a retro-orbital injection of 16 μg on treatment days 6, 15, and 24.

### Gene Expression

Tumor samples harvested at day 20 were homogenized in trizol using a Bead Homogenizer (Bead Ruptor Elite, Omni International). RNA was extracted and isolated using RNeasy Mini Kit (Qiagen, Germany) according to the manufacturer’s instructions and the concentrations were determined using a Nanodrop1000 Spectrophotometer (Thermo Scientific). cDNA was synthesized from total RNA using a QuantiTect Reverse Transcription Kit (Qiagen, Germany). Quantitative real-time polymerase chain reaction (qRT-PCR) was performed using PowerUp SYBR Green qPCR Master Mix (Life Technologies). The Labcyte Echo 550 and MANTIS liquid handling systems were used to load plates to reaction volume of 5 µL. Thermal cycling conditions were performed using the QuantStudio 6 Pro Real-Time PCR System (Applied Biosystems) which included a UDG activation stage at 50°C for 2 min, followed by a DNA polymerase activation stage at 95°C for 2 min followed by 40 cycles of each PCR step (denaturation), 95°C for 15s for annealing/extension and 60°C for 1 min. A melt curve analysis was done to ensure specificity of the corresponding qRT-PCR reactions. For data analysis, the Ct values were exported to an Excel file and fold change was calculated using the ΔΔCt method relative to the expression in the PBS controls (33). *Hprt*, *Pgk1*, and *Tbp* were used as endogenous controls. All reactions were performed in duplicate. Primer information can be found in **Supplemental Table 1**.

### Tumor Cytokine Multiplex Immunoassay

At day 20, tumors were harvested and weighed. Tumor samples (5 μl/mg) were lysed in 20% Cell Lysis Buffer with PMSF (Cell Signaling Technology) and supplemented with Halt Protease and Phosphatase Inhibitor Cocktail (Thermo Scientific). Each tumor was homogenized in bead beater tubes, and the lysate was stored at -80°C. The concentration of 32 cytokines and chemokines in the tumor lysates (MILLIPLEX MAP Mouse Cytokine/Chemokine Magnetic Bead Panel, Millipore) were determined by a multiplex immunoassay following manufacturer’s instructions. The MAGPIX System (Millipore) was used to read the multiplex plate. Concentrations were determined using a standard curve and their respective median fluorescence intensity (MFI) readings (Milliplex Analyst, Millipore). The data underwent log and Z-transformation followed by unbiased hierarchical clustering using Matlab R2019.

### Flow Cytometry

Tumors harvested at day 20 after treatment initiation were processed for flow cytometric analysis as previously described (34). Briefly, tumors were enzymatically dissociated with DNAse and collagenase on a GentleMACS Octodissociator (Miltenyi Biotec) and then filtered through a 70 µm cell strainer. Single cell suspensions were stained with surface antibodies (**Supplemental Table 2**) and then fixed using the eBioscience Foxp3 fixation/permeabilization kit. UltraComp Beads eBeads (Invitrogen) were used for compensation. Flow cytometry was performed on an Attune (ThermoFisher), and compensation matrix and data was analyzed using FlowJo software following published flow cytometry guidelines (35).

### Statistical Analysis

Tumor volume growth curves, displayed as means ± standard error of mean (SEM), were analyzed in a log_10_ transformation and compared between treatment groups using a linear mixed effects model. For survival analysis, Kaplan–Meier curves were generated, and a Log-rank pairwise comparison test with Benjamini-Hochberg (BH) adjustment for p-values was conducted to compare overall survival between treatment groups. To compare the presence of metastatic disease in the lungs at the time of death, Fisher’s exact test followed by a post-hoc pairwise comparison with BH adjustment for p-values was used. A one-way ANOVA followed by a post-hoc multiple comparisons test with Tukey adjustment for p-values was used to determine the statistical significance among cell populations and in gene expression. All analyses were performed in GraphPad Prism or R (v.4.0.2). Adjusted p-values less than 0.05 were considered significant and are indicated in figures as **** = P <0.0001; *** = *P* < 0.001; ** = *P* < 0.01; * = *P* < 0.05.

## Results

### BEMPEG and Anti-CTLA-4 With Local Tumor Treatment Leads to Tumor Regression and Increased Survival in a Single Flank Tumor Model

In mice bearing a flank LLC tumor (~80 mm^3^ on day 1), we tested the efficacy of BEMPEG (16 μg, IV on days 6, 15, 24) and anti-CTLA-4 treatments (200 μg, IP on days 4, 7, 10) combined with local treatment of the tumor through surgical resection (day 16) or delivering three fractions of 8 Gy RT (days 1, 2, 3). When local treatment was combined with BEMPEG and anti-CTLA-4, survival was significantly improved compared to RT or surgery alone (**Figures 1A–D**). BEMPEG and anti-CTLA-4 treatments without local treatment did not improve survival, and these mice died due to primary tumor burden (**Figure 1B**). Combining either BEMPEG or anti-CTLA-4 with RT slightly improved survival over RT alone (p<0.01), but the combination of BEMPEG, anti-CTLA-4, and RT significantly improved survival over either dual treatment (p<0.001, **Figure 1A**). Surgical resection alone only slightly (p<0.05) improved survival, but when combined with anti-CTLA-4 or BEMPEG and anti-CTLA-4, survival was significantly improved (p<0.001, **Figures 1C, D** **)**. At day 90, 33% (4/12) of mice treated with BEMPEG, anti-CTLA-4, and RT were alive, while only 8% (1/12) of mice treated with RT and BEMPEG and 8% (1/12) of mice treated with RT and anti-CTLA-4 remained. An anti-tumor memory response was observed in 67% (2/3) of mice previously treated with BEMPEG, anti-CTLA-4, and RT – as determined by the rejection of re-engraftment with LLC (**Supplemental Figure 2**).

Tumor growth was significantly reduced in mice treated with RT, BEMPEG, and anti-CTLA-4 over dual therapy combinations or monotherapies (p<0.001, **Figure 1E**). At the time of death, lungs from these mice were India ink stained to evaluate metastatic disease. Most mice that received only local treatment to the primary tumor spontaneously developed lung metastases, but when combined with BEMPEG and anti-CTLA-4, this was significantly reduced (p<0.05, **Figures 1F, G** **)**. Mice that received BEMPEG and anti-CTLA-4 treatments or PBS were euthanized at earlier time points due to primary tumor size (**Figure 1H**). Metastatic disease was not found in the lungs of these mice treated with BEMPEG and anti-CTLA-4, however a direct comparison with metastatic rates in the mice treated with RT, BEMPEG, and anti-CTLA-4 was not possible due to the longer time to death in the triple combination group. Note that anti-CTLA-4 and BEMPEG treatments were investigated separately and in combination in a preliminary experiment and did not appear to have a significant impact on tumor growth (**Supplemental Figure 3A**); however, the treatment combination of anti-CTLA-4 and BEMPEG did result in less metastatic disease (**Supplemental Figures 3B-C**).

### BEMPEG and Anti-CTLA-4 Reduce Metastatic Spread in a Disseminated LLC Model

To test the prevention of metastatic disease in a more controlled manner, we tested the use of these combined local and immunotherapies in mice bearing an LLC primary flank tumor (80 mm^3^ on day 1) and disseminated tumor cells administered by IV injection of 2x10^5^ LLC cells at day 10 after treatment initiation. The experiments in **Figure 1** demonstrate that the combination of local control with both anti-CTLA-4 and BEMPEG, and this combination was further investigated in **Figure 2** using a more focused approach. Local control of the primary tumor with three fractions of 8 Gy RT or surgical resection at day 16, when combined with BEMPEG and anti-CTLA-4 treatments, significantly improved survival as compared to local control alone or treatments of BEMPEG and anti-CTLA-4 without local control (p<0.001, **Figures 2A, C** **)**. The mice treated with anti-CTLA-4 and BEMPEG were euthanized due to primary tumor burden, while most mice receiving only local treatment of the primary tumor with RT or surgery died from metastatic disease (**Figures 2B, D** **)**. Again, the combination of BEMPEG and anti-CTLA-4 treatments with RT reduced tumor growth compared to BEMPEG and anti-CTLA-4 or RT alone (p<0.001, **Figure 2E**). At the time of death, in this disseminated metastasis model, significantly fewer mice developed lung tumors when treated with BEMPEG and anti-CTLA-4 than either local treatment alone (p<0.05, **Figures 2F, G** **)**. The addition of BEMPEG and anti-CTLA-4 to local treatment prevented metastatic disease in the lungs as compared to local treatment alone (p<0.001). To further explore the prevention of lung metastases, mice that underwent either surgical resection of the primary tumor or received BEMPEG and anti-CTLA-4 treatments, or both, were euthanized at day 22 and lung metastases were quantified (**Figure 2H**). Mice that received RT, BEMPEG, and anti-CTLA-4 or a combination of these treatments were euthanized at day 35 and lung metastases were quantified (**Figure 2I**). On average, mice that received BEMPEG and anti-CTLA-4 treatments had fewer lung metastases, regardless of local treatment to the tumor (**Figures 2H, I** **)**. Mice treated with RT, BEMPEG, and anti-CTLA-4 had significantly fewer lung metastases than those treated with RT alone or RT and anti-CTLA-4 (p<0.05, **Figure 2I**).

### BEMPEG and Anti-CTLA-4 Combined With Local Radiation Creates a Favorable Adaptive Immune Microenvironment

**Figure 3:**
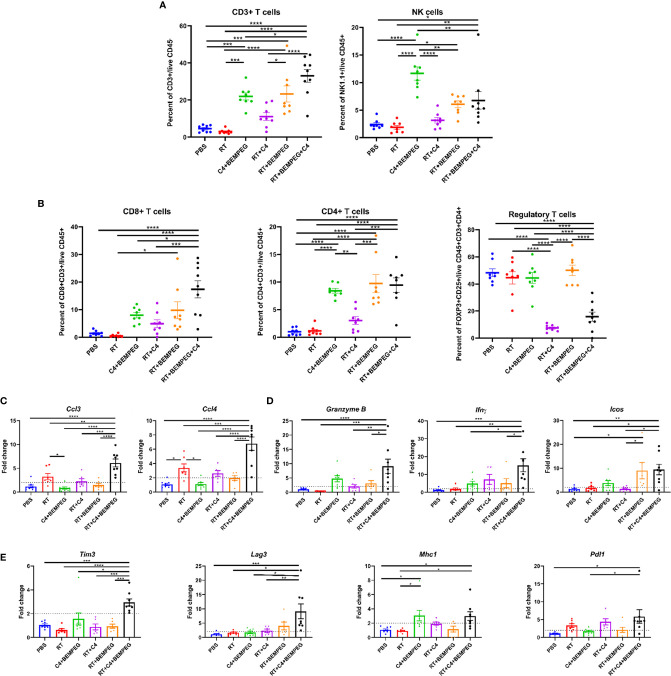
BEMPEG and anti-CTLA-4 combined with local radiation creates a favorable adaptive immune microenvironment. Mice bearing a flank LLC tumor (~80 mm^3^) were given 8 Gy x 3 locally combined with BEMPEG and anti-CTLA-4 (C4) treatments. At day 20 tumors were harvested and processed for qRT-PCR and flow cytometric analyses. **(A, B)** Flow cytometry tumor infiltrates are shown as a percent of parent or grandparent gate. **(C)** Expression of genes associated with immune cell recruitment, **(D)** cytotoxic T lymphocyte activation, and **(E)** immune susceptibility are displayed as fold change in expression relative to the PBS control (mean ± SEM, n≥8). A one-way ANOVA followed by a *post hoc* multiple comparisons test with Tukey adjustments for p-values was used to determine statistical differences among cell populations and gene expression (mean ± SEM, n≥8, **** = P <0.0001; *** = P < 0.001; ** = P < 0.01; * = P < 0.05).

The TME was assessed *via* flow cytometric and qRT-PCR to examine the anti-tumor immune response that we hypothesized to be responsible for the observed tumor regression, improved survival rates, and reduced metastases with these combinations of local therapy and BEMPEG plus anti-CTLA-4. LLC primary tumors were implanted on the right flank. Once average tumor size reached ~80 mm^3^, mice were randomized and treated with combinations of three fractions of 8 Gy RT, BEMPEG, and/or anti-CTLA-4. At day 20 after initial treatment, tumors were dissected. Tumor infiltrating CD3 T cells, NK cells, CD8 T cells, CD4 T cells, and regulatory T cells (Tregs) were quantified using the gating strategy in **Supplemental Figure 4**, which shows a representative dot plot for each treatment group. A significant increase in CD3+ T cells, NK cells, CD8 T cells, and CD4 T cells was found in tumors of mice treated with RT, BEMPEG, and anti-CTLA-4 compared to those treated with RT alone (p<0.01, **Figures 3A, B** **)**. These immunologically “cold” tumors have very low tumor infiltrating CD3 T cells, but CD3 T cells were significantly increased in tumors of mice treated with BEMPEG compared to PBS and RT (**Figure 3A**). The percentage of NK cells out of tumor infiltrating lymphocytes (CD45+) was increased in tumors of mice treated with anti-CTLA-4 and BEMPEG as compared to all other groups (p<0.01, **Figure 3A**). IL2 expands NK cells (20), and NK cell infiltrate was highest in tumors of mice treated with BEMPEG. The percentage of CD8+ T cells out of overall immune cell infiltrate (CD45+) was also increased in tumors of mice treated with RT, BEMPEG, and anti-CTLA-4 over those treated with PBS, RT, anti-CLTA-4 and BEMPEG, or RT and anti-CTLA-4 (p<0.05, **Figure 3B**). CD4+ T cells were increased in tumors from mice treated with BEMPEG compared to PBS, RT, or RT and anti-CTLA-4 (**Figure 3B**). The percentage of immunosuppressive Tregs (CD25+FOXP3+) out of CD4+ T cells was significantly decreased in tumors of mice treated with RT, BEMPEG, and anti-CTLA-4 or RT and anti-CTLA-4 compared to all other groups (p<0.0001, **Figure 3B**).

Using qRT-PCR, transcriptional differences in the bulk tumor mRNA were assessed. *Ccl3* (macrophage inflammatory protein-1α, MIP1α) and *Ccl4* (macrophage inflammatory protein-1β, MIP-1β), genes associated with recruitment and activation of immune cells, were significantly increased in the tumors of mice treated with RT, BEMPEG, and anti-CTLA-4 over all other groups (p<0.01, **Figure 3C**). *Ccl3* has been shown to recruit NK cells to the TME, and *Ccl4* can recruit dermal-resident CD103+ dendritic cells (DCs) (36). Expression of *Granzyme B, Ifnγ* (interferon gamma), and *Icos* (inducible co-stimulator), which are associated with activated cytotoxic T cells, was significantly increased in the tumors of mice treated with RT, BEMPEG, and anti-CTLA-4 over PBS and RT (p<0.05, **Figure 3D**). *Tim3* (T cell immunoglobulin and mucin domain-containing protein 3)*, Lag3* (lymphocyte activation gene 3), *Pdl1* (programmed death ligand 1), and *Mhc1* (major histocompatibility complex-1), which are genes that regulate tumor cell immune susceptibility, were significantly increased in the tumors of mice treated with RT, BEMPEG, and anti-CTLA-4 (**Figure 3E**). *Pdl1* expression was significantly increased in the tumors of mice treated with RT, BEMPEG, and anti-CTLA-4 compared to mice treated with PBS or BEMPEG and anti-CTLA-4 (p<0.05). *Mhc1* expression was significantly increased in the tumors of mice treated with RT, BEMPEG, and anti-CTLA-4 or BEMPEG and anti-CTLA-4 compared to mice treated with PBS or RT (p<0.05).

### Radiation, Anti-CTLA-4, and BEMPEG Combined Treatments Change the Cytokine Profiles of the TME

**Figure 4:**
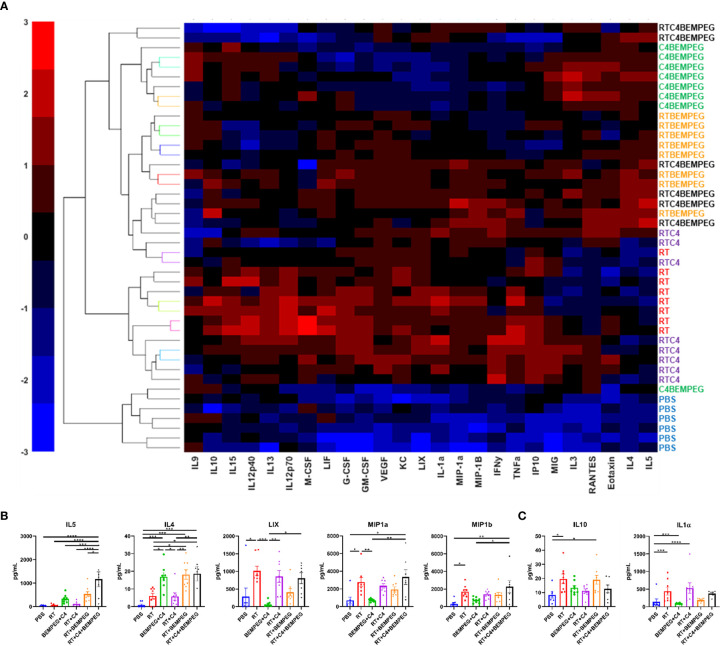
Radiation, anti-CTLA-4, and BEMPEG combined treatments change the cytokine profiles of the TME. Mice bearing a flank LLC tumor (~80 mm^3^) were given 8 Gy x 3 locally combined with BEMPEG and anti-CTLA-4 (C4) treatments. At day 20 tumors were collected and lysed. Multiplex immunoassay analysis was performed to determine concentrations of 25 cytokines and chemokines. **(A)** Unbiased hierarchal clustering was used to sort tumors based on detected levels of cytokines and chemokines. **(B, C)** A one-way ANOVA followed by a Tukey multiple comparisons test was used to determine statistical differences among cell populations, (mean ± SEM, n≥6, **** = P <0.0001; *** = P < 0.001; ** = P < 0.01; * = P < 0.05). **(B)** Concentrations of immune stimulating cytokines and **(C)** cytokines associated with immunosuppressive or inhibitory functions are shown.

Further examination of the TME was performed using tumor fragments from these same mice as above by analyzing the concentrations of cytokines and chemokines in the TME. A multiplex cytokine assay was performed, and unsupervised hierarchal clustering was used to sort tumors based on detected levels of 25 cytokines and chemokines (**Figure 4A**, and **Supplemental Figure 5**). PBS samples had low levels of all markers and clustered together. Tumors from mice treated with BEMPEG in combination with RT, anti-CTLA-4, or both also clustered together. Significant differences were observed in several immune stimulating cytokines with the treatment of RT, anti-CTLA-4, and BEMPEG, including MIP1α, MIP1β, LIX, IL4, and IL5 (p<0.05, **Figure 4B**). IL-10 and IL-1α, cytokines associated with immunosuppressive or inhibitory functions, were significantly increased in tumors treated with RT alone compared to PBS (p<0.05), but not significantly increased in tumors from mice given the combination treatment of RT, anti-CTLA-4, and BEMPEG (p>0.05, **Figure 4C**).

## Discussion

In a spontaneously metastasizing immunologically “cold” model of early-stage NSCLC, we report that the combination of anti-CTLA-4 and BEMPEG inhibits the development of distant metastases and effectively eradicates IV injected tumor cells representing micro-metastatic disease. When combined with an effective local treatment (hypo-fractionated RT or surgery), this combination enables durable complete primary tumor response, long-term disease-free survival, and evidence of anti-tumor immune memory. While BEMPEG and anti-CTLA-4 alone do not control established primary tumors in this model, this combination of immunotherapy does augment local tumor control when combined with moderate dose hypo-fractionated RT or surgery. Similarly, with only primary tumor control, mice succumb to metastatic disease unless combined with BEMPEG and anti-CTLA-4. With recent studies showing safety for the combination of BEMPEG and anti-CTLA-4 or other ICIs in non-human primates (25) and humans with metastatic solid tumors (23), our findings highlight the exciting translational potential for testing the capacity of this treatment combination to improve the cure rates for patients receiving locally directed treatments for early-stage NSCLC and potentially other localized cancers with high-risk for occult metastatic disease.

Given the prominent role of Tregs in suppressing anti-tumor immunity in NSCLC and other tumor types (20, 26–28), we hypothesized that the efficacy of BEMPEG and anti-CTLA-4 in controlling micrometastatic NSCLC might result from overcoming Treg-mediated immune suppression with anti-CTLA-4 (37) and selectively stimulating clonal expansion of effector lymphocytes but not Tregs with BEMPEG (24). Our data from this immunologically “cold” LLC murine model of micrometastatic NSCLC supports these potential mechanisms. Immunologically “cold” tumors have few tumor infiltrating lymphocytes (TILs) as seen in the flow analysis of the CD3+ T cells in the PBS and RT alone controls (**Figure 3**). However, the composition of tumor infiltrating immune cells in these tumors was modified when BEMPEG or anti-CTLA-4 were added to local treatments. Specifically, we observed increased levels of CD8+ and CD4+ T cells as well as NK cells in tumor specimens following RT+BEMPEG, as compared to RT alone (**Figure 3**). We further confirmed a reduction of tumor infiltrating Tregs with the addition of anti-CTLA-4 to RT+BEMPEG, as compared to RT+BEMPEG alone (**Figure 3**).

Anti-CTLA4 has been shown to deplete Tregs *via* antibody-dependent cell-mediated cytotoxicity (ADCC) (37). Consistent with this, we observed that mice treated with RT and anti-CTLA4 with or without BEMPEG had significantly fewer Tregs in the TME than all other treatment groups, which suggests anti-CTLA-4 maintains a role in the depletion of Tregs in these combination treatment approaches (**Figure 3B**). Additionally, BEMPEG has been reported to selectively expand populations conventional effector T cells with relatively reduced effect on Tregs (29). Here, we also observe that mice treated with BEMPEG (anti-CTLA-4+BEMPEG, RT+BEMPEG, and RT+anti-CTLA-4+BEMPEG) had tumors with significantly increased infiltration by CD4 but not Tregs (CD25+FOXP3+CD4+ T cells) (**Figure 3B**). Mice treated with RT+BEMPEG or RT+BEMPEG+anti-CTLA-4 had significantly more CD8 T cells compared to the RT control. BEMPEG has been shown to increase CD8 T cells in the TME (29, 38). Together, this data suggests that the therapeutic mechanisms of anti-CTLA4 and BEMPEG are maintained and non-redundant when combined and delivered in conjunction with locally directed therapies like surgery or RT. This can provide a two-pronged approach to activating anti-tumor immunity against immunologically cold tumors by depleting immunosuppressive Tregs and driving increased infiltration by effector CD8 T cells in the TME. Our data also demonstrate that BEMPEG may increase NK cell recruitment to the TME, as all treatment groups with BEMPEG had increased NK cells compared to the RT and PBS controls (**Figure 3**). In other tumor models, studies have found that BEMPEG can increase NK cells in the peripheral blood (38) and in the tumor (25). However, *Ccl3* expression, which is associated with NK cell recruitment, did not follow this same trend. Future experiments should further investigate the mechanisms BEMPEG-induced recruitment of NK cells to the TME.

In addition to inducing an effector-dominated lymphocytic immune infiltrate in tumors, we observe that BEMPEG and anti-CTLA-4 increased the activation of tumor infiltrating immune cells, as measured by the increased expression of genes associated with cytotoxic T lymphocyte activation (*Icos*, *Granzyme B*, and *Ifnγ*) (**Figure 3**) and increased production of several immune stimulatory cytokines in the primary tumor microenvironment (**Figure 4**) following combined RT, BEMPEG, and anti-CTLA-4, as compared to RT alone. Concurrent with these effects, we observed that combined BEMPEG and anti-CTLA-4 stimulated increased expression of *Mhc1* as well as upregulation of additional immune checkpoint ligands (*Pdl1*, *Tim3*, and *Lag3*). These expression changes may be secondary to the increased IFN*γ* production that we observed in the tumor microenvironment (39–41). In future studies it may be valuable to test this treatment approach in combination with inhibitors targeting these additional checkpoint ligands to further enhance the magnitude and/or duration of anti-tumor immunity stimulated by BEMPEG and anti-CTLA-4.

Our results support and expand upon several recently reported preclinical studies including one demonstrating that BEMPEG and ICIs synergize to augment T cell mediated anti-cancer immunity (29). A separate recent study suggested a cooperative therapeutic interaction between RT and BEMPEG in immunogenic tumor models, which sometimes exhibited strong response to BEMPEG alone (38). That study reported that combined RT and BEMPEG triggered an expanded CD8 T cell infiltrate. Here, we observe a similar effect and demonstrate that it may be further advanced by combination with anti-CTLA-4. Preclinical studies demonstrate that systemic effects of RT in priming a systemic anti-tumor T cell response are achieved more reliably when RT is combined with anti-CTLA-4 and/or anti-PD-1/PD-L1 checkpoint blockade (42, 43). A recent prospective single arm clinical study evaluating RT and anti-CTLA-4 in patients with NSCLC demonstrated safety and in favorably responding patients showed that RT-induced T cell recognition of tumor-specific neo-antigens (19).

Clinical studies are already underway integrating anti-PD1/PD-L1 into neo-adjuvant or adjuvant strategies for the treatment of early-stage NSCLC (e.g., NCT02504372, NCT02998528, NCT04025879, and NCT04214262). Given the response rates to anti-PD1/anti-PD-L1 therapies in other studies of patients with NSCLC, our expectation is that this approach will be effective in preventing metastatic progression for some, but not all early-stage NSCLC patients. As biomarkers emerge that can predict response to neo-adjuvant or adjuvant treatments with anti-PD1/PDL1, additional approaches will be needed for patients with tumors that are not responsive. The data we present here suggest that a combination of BEMPEG and anti-CTLA-4 with local control could be an effective alternative treatment option for patients with NSCLC that is not responsive to anti-PD1/PDL1 therapies, and clinical investigation is warranted to test this. LLC may be a good model for this type of disease, as it is immunologically “cold” and does not respond to anti-PDL1 (44).

While we investigated a model of early-stage localized cancer with occult micro-metastases, our findings may also have implications for advanced metastatic disease. We observe that combinations of anti-CTLA-4 and BEMPEG are powerful in eradicating micro-metastases but do not adequately control well-established macroscopic tumor sites. Rather, such tumors require locally directed therapies. Nearly all patients with metastatic NSCLC have more than one macroscopic tumor site. It is beyond the scope of the present study to evaluate whether targeting one tumor site with RT would prime effective anti-tumor immunity against other well-established macroscopic metastases or whether it would be beneficial to deliver local RT to all such tumor sites to achieve curative response when combined with anti-CTLA-4 and BEMPEG. Such approaches delivering RT to all tumor sites are increasingly practiced in patients with oligometastatic disease (45, 46). In settings of widespread metastatic disease this may be achieved using targeted radionuclide therapies (47, 48). In future studies, it will be interesting to test these approaches to combining radiation therapy with BEMPEG and ICIs in the treatment of macro-metastatic disease.

We acknowledge several weaknesses in this study including the exclusive use of heterotopic transplantable syngeneic murine models. Such models have clear value in translational immuno-oncology research because they enable *in vivo* hypothesis testing in settings of intact host immunity. However, it is understood that certain immune and radiobiological mechanisms in mice and heterotopic transplantable tumors may differ from those observed in human immune systems and tumors. Therefore, additional preclinical and clinical studies will be needed to establish the translational potential of the treatment approach developed here. In addition, the doses and conformality of RT delivered in this study do not directly replicate approaches used clinically for treatment of early-stage NSCLC. Specifically, we employ a hypofractionated dose of RT that has been reported to be optimal for activating a type I interferon response (49, 50). While similar, this regimen delivers a lower biologically equivalent dose than the stereotactic body RT (SBRT) regimens that are commonly employed clinically for treatment of early-stage NSCLC. Notably, for animal safety reasons we are not able to deliver the high dose SBRT clinical regimens to mice.

Despite such limitations, our results provide rationale for further preclinical and clinical testing of the combination of BEMPEG and anti-CTLA-4 together with local treatments like surgery or RT. Although testing of novel therapeutic combinations in cancer commonly begins in metastatic settings, our findings highlight an opportunity to potentially improve the treatment of high-risk early-stage cancers such as NSCLC. We advocate for increased testing of such novel treatments in these early stage patient populations with potentially curative cancers – a setting in which the impact of these therapeutic innovations may be greatest (51).

## Data Availability Statement

The original contributions presented in the study are included in the article/**Supplementary Material**, further inquiries can be directed to the corresponding authors.

## Ethics Statement

The animal study was reviewed and approved by University of Wisconsin Institutional Animal Care and Use Committee.

## Author Contributions

RB, AB, and RP designed and performed experiments and analysed data. AP, LZ, IA, PMC, GS, PAC, and RS also performed experiments. ZM and RP contributed to experimental design and supervised the project. AB, TL, and KK analysed data. AB wrote the manuscript with input from all authors. All authors contributed to the article and approved the submitted version.

## Funding

The authors’ work is supported in part by grants from NIH P30 CA014520, NIH 1DP5OD024576, NIH U01CA233102, F30CA228315, Radiological Society of North America Research Fellow Grant RF1716, the Shaw Scientist Award, American Society of Clinical Oncology Hayden Family Foundation Young Investigator Award 12805, and K08CA241319. RP was supported in part by the Hillman Cancer Center Early Career Fellowship for Innovative Cancer Research and the Bentson Translational Research Fellowship.

## Conflict of Interest

The authors declare that the research was conducted in the absence of any commercial or financial relationships that could be construed as a potential conflict of interest.

## References

[1] Infante M Cavuto S Lutman FR Passera E Chiarenza M Chiesa G. Long-Term Follow-up Results of the DANTE Trial, a Randomized Study of Lung Cancer Screening with Spiral Computed Tomography. Am J Respir Crit Care Med (2015) 191(10):1166–75. 10.1164/rccm.201408-1475OC 25760561

[2] Pastorino U Rossi M Rosato V Marchiano A Sverzellati N Morosi C. Annual or biennial CT screening versus observation in heavy smokers: 5-year results of the MILD trial. Eur J Cancer Prev (2012) 21(3):308–15. 10.1097/CEJ.0b013e328351e1b6 22465911

[3] National Lung Screening Trial Research T Aberle DR Adams AM Berg CD Black WC Clapp JD. Reduced lung-cancer mortality with low-dose computed tomographic screening. N Engl J Med (2011) 365(5):395–409. 10.1056/NEJMoa1102873 21714641 PMC4356534

[4] Saghir Z Dirksen A Ashraf H Bach KS Brodersen J Clementsen PF. CT screening for lung cancer brings forward early disease. The randomised Danish Lung Cancer Screening Trial: status after five annual screening rounds with low-dose CT. Thorax (2012) 67(4):296–301. 10.1136/thoraxjnl-2011-200736 22286927

[5] Higuchi M Yaginuma H Yonechi A Kanno R Ohishi A Suzuki H. Long-term outcomes after video-assisted thoracic surgery (VATS) lobectomy versus lobectomy via open thoracotomy for clinical stage IA non-small cell lung cancer. J Cardiothorac Surg (2014) 9:88. 10.1186/1749-8090-9-88 24886655 PMC4058716

[6] Sakuraba M Miyamoto H Oh S Shiomi K Sonobe S Takahashi N. Video-assisted thoracoscopic lobectomy vs. conventional lobectomy via open thoracotomy in patients with clinical stage IA non-small cell lung carcinoma. Interact Cardiovasc Thorac Surg (2007) 6(5):614–7. 10.1510/icvts.2007.157701 17670728

[7] Shiraishi T Shirakusa T Hiratsuka M Yamamoto S Iwasaki A. Video-assisted thoracoscopic surgery lobectomy for c-T1N0M0 primary lung cancer: its impact on locoregional control. Ann Thorac Surg (2006) 82(3):1021–6. 10.1016/j.athoracsur.2006.04.031 16928528

[8] Fakiris AJ McGarry RC Yiannoutsos CT Papiez L Williams M Henderson MA. Stereotactic body radiation therapy for early-stage non-small-cell lung carcinoma: four-year results of a prospective phase II study. Int J Radiat Oncol Biol Phys (2009) 75(3):677–82. 10.1016/j.ijrobp.2008.11.042 19251380

[9] Timmerman R Paulus R Galvin J Michalski J Straube W Bradley J. Stereotactic body radiation therapy for inoperable early stage lung cancer. JAMA (2010) 303(11):1070–6. 10.1001/jama.2010.261 PMC2907644 20233825

[10] Videtic GM Hu C Singh AK Chang JY Parker W Olivier KR. A Randomized Phase 2 Study Comparing 2 Stereotactic Body Radiation Therapy Schedules for Medically Inoperable Patients With Stage I Peripheral Non-Small Cell Lung Cancer: NRG Oncology RTOG 0915 (NCCTG N0927). Int J Radiat Oncol Biol Phys (2015) 93(4):757–64. 10.1016/j.ijrobp.2015.07.2260 PMC4744654 26530743

[11] Siegel RL Miller KD Jemal A. Cancer statistics, 2019. CA Cancer J Clin (2019) 69(1):7–34. 10.3322/caac.21551 30620402

[12] Peterson J Niles C Patel A Boujaoude Z Abouzgheib W Goldsmith B. Stereotactic Body Radiotherapy for Large (> 5 cm) Non-Small-Cell Lung Cancer. Clin Lung Cancer (2017) 18(4):396–400. 10.1016/j.cllc.2016.11.020 28040379

[13] Verma V Shostrom VK Kumar SS Zhen W Hallemeier CL Braunstein SE. Multi-institutional experience of stereotactic body radiotherapy for large (>/=5 centimeters) non-small cell lung tumors. Cancer (2017) 123(4):688–96. 10.1002/cncr.30375 PMC10905610 27741355

[14] Mok TSK Wu YL Kudaba I Kowalski DM Cho BC Turna HZ. Pembrolizumab versus chemotherapy for previously untreated, PD-L1-expressing, locally advanced or metastatic non-small-cell lung cancer (KEYNOTE-042): a randomised, open-label, controlled, phase 3 trial. Lancet (2019) 393(10183):1819–30. 10.1016/S0140-6736(18)32409-7 30955977

[15] Gandhi L Rodriguez-Abreu D Gadgeel S Esteban E Felip E De Angelis F. Pembrolizumab plus Chemotherapy in Metastatic Non-Small-Cell Lung Cancer. N Engl J Med (2018) 378(22):2078–92. 10.1056/NEJMoa1801005 29658856

[16] Garon EB Rizvi NA Hui R Leighl N Balmanoukian AS Eder JP. Pembrolizumab for the treatment of non-small-cell lung cancer. N Engl J Med (2015) 372(21):2018–28. 10.1056/NEJMoa1501824 25891174

[17] Paz-Ares L Luft A Vicente D Tafreshi A Gumus M Mazieres J. Pembrolizumab plus Chemotherapy for Squamous Non-Small-Cell Lung Cancer. N Engl J Med (2018) 379(21):2040–51. 10.1056/NEJMoa1810865 30280635

[18] Reck M Rodriguez-Abreu D Robinson AG Hui R Csoszi T Fulop A. Pembrolizumab versus Chemotherapy for PD-L1-Positive Non-Small-Cell Lung Cancer. N Engl J Med (2016) 375(19):1823–33. 10.1056/NEJMoa1606774 27718847

[19] Formenti SC Rudqvist NP Golden E Cooper B Wennerberg E Lhuillier C. Radiotherapy induces responses of lung cancer to CTLA-4 blockade. Nat Med (2018) 24(12):1845–51. 10.1038/s41591-018-0232-2 PMC6286242 30397353

[20] Boyman O Sprent J. The role of interleukin-2 during homeostasis and activation of the immune system. Nat Rev Immunol (2012) 12(3):180–90. 10.1038/nri3156 22343569

[21] Davar D Ding F Saul M Sander C Tarhini AA Kirkwood JM. High-dose interleukin-2 (HD IL-2) for advanced melanoma: a single center experience from the University of Pittsburgh Cancer Institute. J Immunother Cancer (2017) 5(1):74. 10.1186/s40425-017-0279-5 28923120 PMC5604296

[22] Atkins MB Lotze MT Dutcher JP Fisher RI Weiss G Margolin K. High-dose recombinant interleukin 2 therapy for patients with metastatic melanoma: analysis of 270 patients treated between 1985 and 1993. J Clin Oncol (1999) 17(7):2105–16. 10.1200/JCO.1999.17.7.2105 10561265

[23] Diab A Tannir NM Bentebibel SE Hwu P Papadimitrakopoulou V Haymaker C. Bempegaldesleukin (NKTR-214) plus Nivolumab in Patients with Advanced Solid Tumors: Phase I Dose-Escalation Study of Safety, Efficacy, and Immune Activation (PIVOT-02). Cancer Discovery (2020) 10:1158–73. 10.1158/2159-8290.CD-19-1510 32439653

[24] Charych D Khalili S Dixit V Kirk P Chang T Langowski J. Modeling the receptor pharmacology, pharmacokinetics, and pharmacodynamics of NKTR-214, a kinetically-controlled interleukin-2 (IL2) receptor agonist for cancer immunotherapy. PloS One (2017) 12(7):e0179431. 10.1371/journal.pone.0179431 28678791 PMC5497954

[25] Charych DH Hoch U Langowski JL Lee SR Addepalli MK Kirk PB. NKTR-214, an Engineered Cytokine with Biased IL2 Receptor Binding, Increased Tumor Exposure, and Marked Efficacy in Mouse Tumor Models. Clin Cancer Res an Off J Am Assoc Cancer Res (2016) 22(3):680–90. 10.1158/1078-0432.CCR-15-1631 26832745

[26] Wang X Rickert M Garcia KC. Structure of the quaternary complex of interleukin-2 with its alpha, beta, and gammac receptors. Science (2005) 310(5751):1159–63. 10.1126/science.1117893 16293754

[27] Petersen RP Campa MJ Sperlazza J Conlon D Joshi MB Harpole DH Jr.. Tumor infiltrating Foxp3+ regulatory T-cells are associated with recurrence in pathologic stage I NSCLC patients. Cancer (2006) 107(12):2866–72. 10.1002/cncr.22282 17099880

[28] Shimizu K Nakata M Hirami Y Yukawa T Maeda A Tanemoto K. Tumor-infiltrating Foxp3+ regulatory T cells are correlated with cyclooxygenase-2 expression and are associated with recurrence in resected non-small cell lung cancer. J Thorac Oncol (2010) 5(5):585–90. 10.1097/JTO.0b013e3181d60fd7 20234320

[29] Sharma M Khong H Fa’ak F Bentebibel SE Janssen LME Chesson BC. Bempegaldesleukin selectively depletes intratumoral Tregs and potentiates T cell-mediated cancer therapy. Nat Commun (2020) 11(1):661. 10.1038/s41467-020-14471-1 32005826 PMC6994577

[30] Bentebibel SE Hurwitz ME Bernatchez C Haymaker C Hudgens CW Kluger HM. A First-in-Human Study and Biomarker Analysis of NKTR-214, a Novel IL2Rbetagamma-Biased Cytokine, in Patients with Advanced or Metastatic Solid Tumors. Cancer Discovery (2019) 9(6):711–21. 10.1158/2159-8290.CD-18-1495 30988166

[31] Wong RJ Chan MK Yu Z Kim TH Bhargava A Stiles BM. Effective intravenous therapy of murine pulmonary metastases with an oncolytic herpes virus expressing interleukin 12. Clin Cancer Res an Off J Am Assoc Cancer Res (2004) 10(1 Pt 1):251–9. 10.1158/1078-0432.CCR-0197-3 14734477

[32] Inoue M Nakashima R Enomoto M Koike Y Zhao X Yip K. Plasma redox imbalance caused by albumin oxidation promotes lung-predominant NETosis and pulmonary cancer metastasis. Nat Commun (2018) 9(1):5116. 10.1038/s41467-018-07550-x 30504805 PMC6269536

[33] Livak KJ Schmittgen TD. Analysis of relative gene expression data using real-time quantitative PCR and the 2(-Delta Delta C(T)) Method. Methods (2001) 25(4):402–8. 10.1006/meth.2001.1262 11846609

[34] Patel RB Ye M Carlson PM Jaquish A Zangl L Ma B. Development of an In Situ Cancer Vaccine via Combinational Radiation and Bacterial-Membrane-Coated Nanoparticles. Adv Mater (2019) 31(43):e1902626. 10.1002/adma.201902626 31523868 PMC6810793

[35] Maecker HT Trotter J. Flow cytometry controls, instrument setup, and the determination of positivity. Cytometry A (2006) 69(9):1037–42. 10.1002/cyto.a.20333 16888771

[36] Allen F Bobanga ID Rauhe P Barkauskas D Teich N Tong C. CCL3 augments tumor rejection and enhances CD8(+) T cell infiltration through NK and CD103(+) dendritic cell recruitment via IFNgamma. Oncoimmunology (2018) 7(3):e1393598. 10.1080/2162402X.2017.1393598 29399390 PMC5790335

[37] Selby MJ Engelhardt JJ Quigley M Henning KA Chen T Srinivasan M. Anti-CTLA-4 antibodies of IgG2a isotype enhance antitumor activity through reduction of intratumoral regulatory T cells. Cancer Immunol Res (2013) 1(1):32–42. 10.1158/2326-6066.CIR-13-0013 24777248

[38] Walker JM Rolig AS Charych DH Hoch U Kasiewicz MJ Rose DC. NKTR-214 immunotherapy synergizes with radiotherapy to stimulate systemic CD8(+) T cell responses capable of curing multi-focal cancer. J Immunother Cancer (2020) 8(1):e000464. 10.1136/jitc-2019-000464 32457127 PMC7252958

[39] Philippou Y Sjoberg HT Murphy E Alyacoubi S Jones KI Gordon-Weeks AN. Impacts of combining anti-PD-L1 immunotherapy and radiotherapy on the tumour immune microenvironment in a murine prostate cancer model. Br J Cancer (2020) 123(7):1089–100. 10.1038/s41416-020-0956-x PMC7525450 32641865

[40] Lugade AA Sorensen EW Gerber SA Moran JP Frelinger JG Lord EM. Radiation-induced IFN-gamma production within the tumor microenvironment influences antitumor immunity. J Immunol (2008) 180(5):3132–9. 10.4049/jimmunol.180.5.3132 18292536

[41] Gerber SA Sedlacek AL Cron KR Murphy SP Frelinger JG Lord EM. IFN-gamma mediates the antitumor effects of radiation therapy in a murine colon tumor. Am J Pathol (2013) 182(6):2345–54. 10.1016/j.ajpath.2013.02.041 PMC3668027 23583648

[42] Pilones KA Vanpouille-Box C Demaria S. Combination of radiotherapy and immune checkpoint inhibitors. Semin Radiat Oncol (2015) 25(1):28–33. 10.1016/j.semradonc.2014.07.004 25481263

[43] Vanpouille-Box C Pilones KA Wennerberg E Formenti SC Demaria S. In situ vaccination by radiotherapy to improve responses to anti-CTLA-4 treatment. Vaccine (2015) 33(51):7415–22. 10.1016/j.vaccine.2015.05.105 PMC4684480 26148880

[44] Chen JL Pan CK Huang YS Tsai CY Wang CW Lin YL. Evaluation of antitumor immunity by a combination treatment of high-dose irradiation, anti-PDL1, and anti-angiogenic therapy in murine lung tumors. Cancer Immunol Immunother (2021) 70(2):391–404. 10.1007/s00262-020-02690-w 32761424 PMC10991177

[45] Tsao MN Ven LI Cheung P Poon I Ung Y Louie AV. Stereotactic Body Radiation Therapy for Extracranial Oligometastatic Non-small-cell Lung Cancer: A Systematic Review. Clin Lung Cancer (2020) 21(2):95–105 e1. 10.1016/j.cllc.2019.11.007 31959533

[46] Palma DA Olson R Harrow S Correa RJM Schneiders F Haasbeek CJA. Stereotactic ablative radiotherapy for the comprehensive treatment of 4-10 oligometastatic tumors (SABR-COMET-10): study protocol for a randomized phase III trial. BMC Cancer (2019) 19(1):816. 10.1186/s12885-019-5977-6 31426760 PMC6699121

[47] Jagodinsky JC Morris ZS. Priming and Propagating Anti-tumor Immunity: Focal Hypofractionated Radiation for in Situ Vaccination and Systemic Targeted Radionuclide Theranostics for Immunomodulation of Tumor Microenvironments. Semin Radiat Oncol (2020) 30(2):181–6. 10.1016/j.semradonc.2019.12.008 PMC7286051 32381297

[48] Sgouros G Bodei L McDevitt MR Nedrow JR. Radiopharmaceutical therapy in cancer: clinical advances and challenges. Nat Rev Drug Discovery (2020) 19(9):589–608. 10.1038/s41573-020-0073-9 32728208 PMC7390460

[49] Dewan MZ Galloway AE Kawashima N Dewyngaert JK Babb JS Formenti SC. Fractionated but not single-dose radiotherapy induces an immune-mediated abscopal effect when combined with anti-CTLA-4 antibody. Clin Cancer Res an Off J Am Assoc Cancer Res (2009) 15(17):5379–88. 10.1158/1078-0432.CCR-09-0265 PMC2746048 19706802

[50] Vanpouille-Box C Alard A Aryankalayil MJ Sarfraz Y Diamond JM Schneider RJ. DNA exonuclease Trex1 regulates radiotherapy-induced tumour immunogenicity. Nat Commun (2017) 8:15618. 10.1038/ncomms15618 28598415 PMC5472757

[51] Morris ZS Harari PM. Interaction of radiation therapy with molecular targeted agents. J Clin Oncol (2014) 32(26):2886–93. 10.1200/JCO.2014.55.1366 PMC4152717 25113770

