# Supplementary material for: Combination of Bempegaldesleukin and Anti-CTLA-4 Prevents Metastatic Dissemination After Primary Resection or Radiotherapy in a Preclinical Model of Non-Small Cell Lung Cancer
Source: Front Oncol. 2021 Apr 15;11:645352. doi: 10.3389/fonc.2021.645352 (PMC8083981; doi:10.3389/fonc.2021.645352)
Supplement: Supplementary file 1 [file DataSheet_1.docx]

Supplemental figures and tables:

| 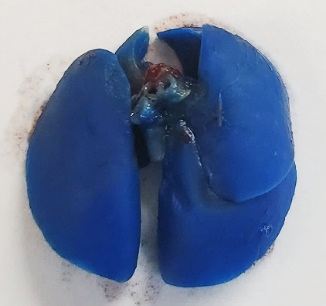  **A** | 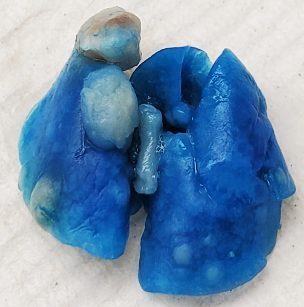 | 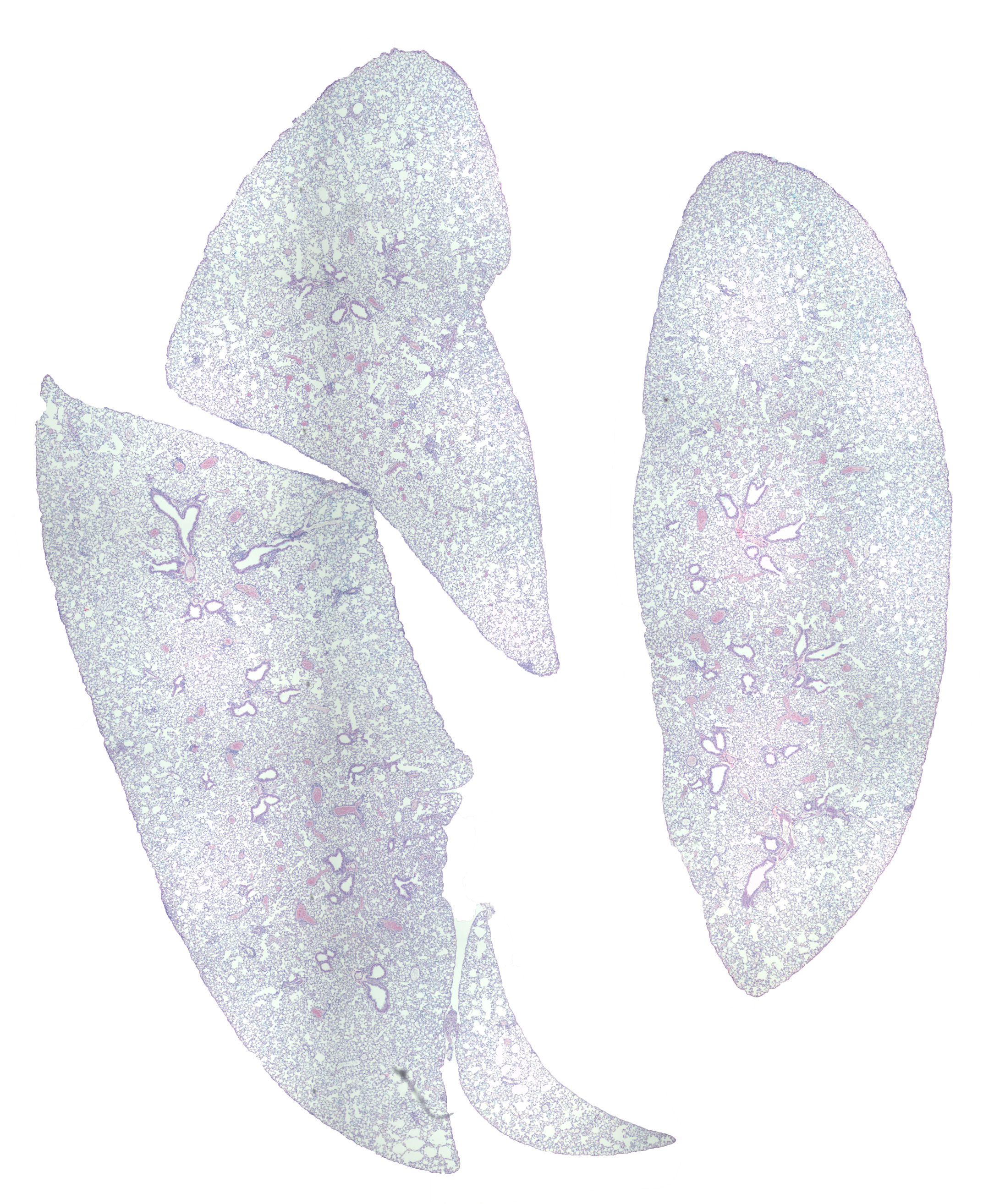  **B** | 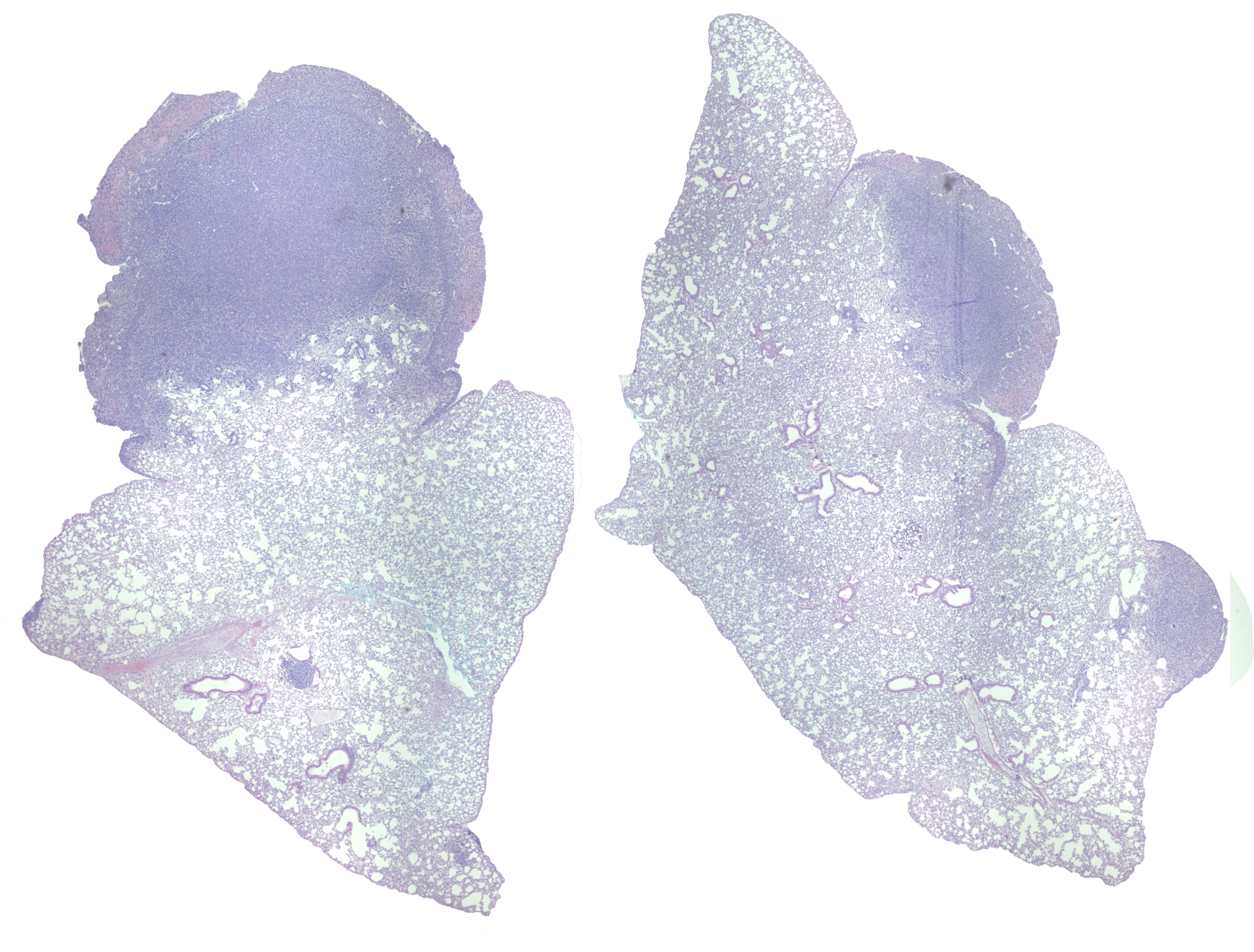 |
| --- | --- | --- | --- |

Supplemental Figure 1. A) Representative India ink-stained healthy lungs on the left compared to lungs with metastatic disease on the right are displayed. B) Representative H&E-stained sections of healthy lungs on the left compared to lungs with metastatic disease on the right are shown.

| Gene | Forward Primer | Reverse Primer |
| --- | --- | --- |
| *Ccl3* | ACATCATGAAGGTCTCCACCAC | CCATATGGCGCTGAGAAGACT |
| *Ccl4* | CCTCCCACTTCCTGCTGTTT | GTCTCATAGTAATCCATCACAAAGC |
| *Granzyme B* | AAAGGCAGGGGAGATCATCG | AGGCTGCTGATCCTTGATCG |
| *Ifnγ* | AGCGGCTGACTGAACTCAGATTGTAG | GTCACAGTTTTCAGCTGTATAGGG |
| *Icos* | TCTTGTAAATACCCTGAGACTGTCC | TCGCAGAGGACTTCTCTCTCTC |
| *Lag3* | CAGCTCAATGCCACTGTCAC | TTTCCAGATGCCGGGGTTAC |
| *Tim3* | TGTGCTCAAGGGGAACTGAC | CCCAGTCACCGGTTGTTCTT |
| *Mhc-I (H2kb)* | AGAAGTGGGCATCTGTGGTG | GACAACCAGAACAGCAACGG |
| *Pdl1* | ATGTCAGGCCGAGGGTTATC | TCTCTTCCCACTCACGGGTT |
| *Hprt* | AGCCTAAGATGAGCGCAAGT | GGCCCACAGGACTAGAACACC |
| *Pgk1* | GGCATTCTGCACGCTTCAAA | CGACATTTTGGCAACACCGT |
| *Tbp1* | GTTGGGCTTCCCAGCTAAGT | CACAAGGCCTTCCAGCCTTA |

Supplemental Table 1. Primer sequences used.

|  | Target | Fluorophore | Company (Cat.) | Clone |
| --- | --- | --- | --- | --- |
| Live/Dead | | GhostRed 780 | Tonbo | - |
| CD45 | | BV510 | BioLegend | 30-F11 |
| CD3 | | APC | BioLegend | 145-2C11 |
| CD4 | | BV785 | BioLegend | GK1.5 |
| CD8 | | APC-R700 | BD Biosciences | 53-6.7 |
| NK1.1 | | PE-CF594 | BD Biosciences | PK136 |
| CD25 | | BB515 | BD Biosciences | PC61 |
| FOXP3 | | PE-Cy7 | Invitrogen | FJK-16s |

Supplemental Table 2. Flow cytometry antibodies used to stain cells in disaggregated tumors.


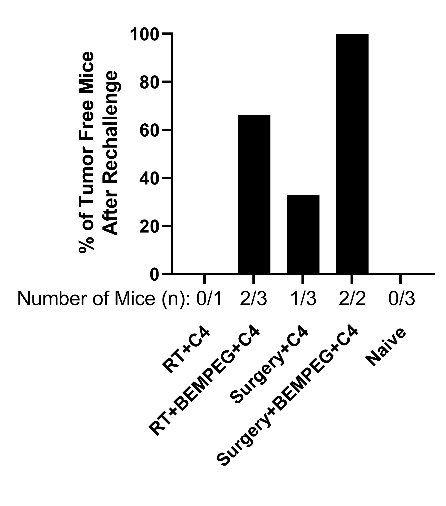

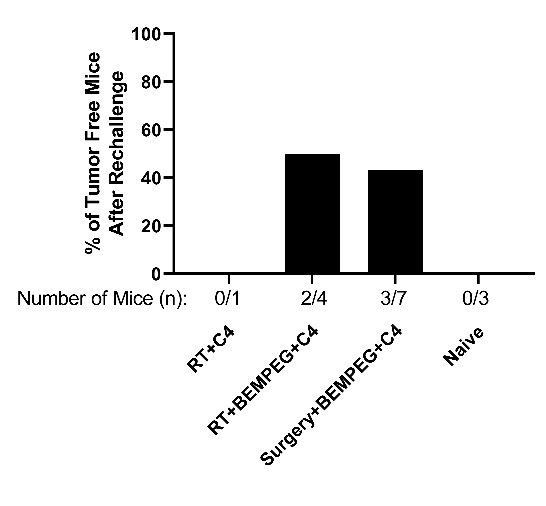


**B**

**A**

Supplemental Figure 2. After 90 days, mice with a complete tumor response were rechallenged to evaluate for immune memory by an additional injection of 1 x 10^6^ LLC cells intradermally in the opposite (left) flank. Age-matched naive mice were injected in the left flank with the same number of tumor cells. A) Mice that previously rejected a primary tumor as seen in Figure 1. B) Mice that previously rejected a primary tumor and IV delivered tumor cells as seen in Figure 2. No significant differences.

| 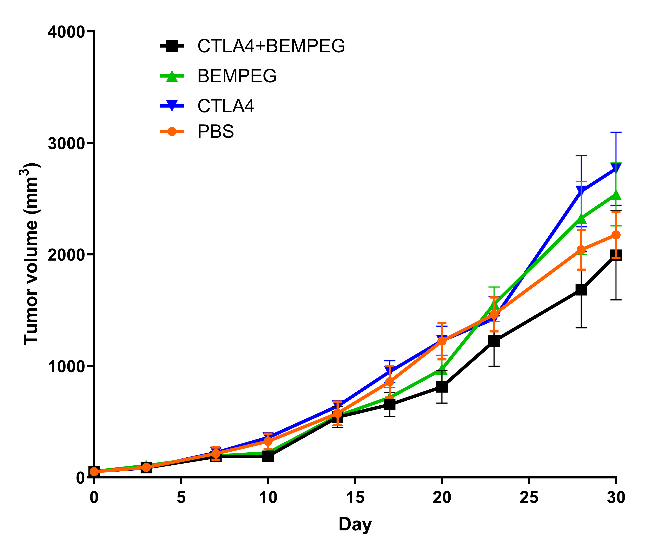  **A** | 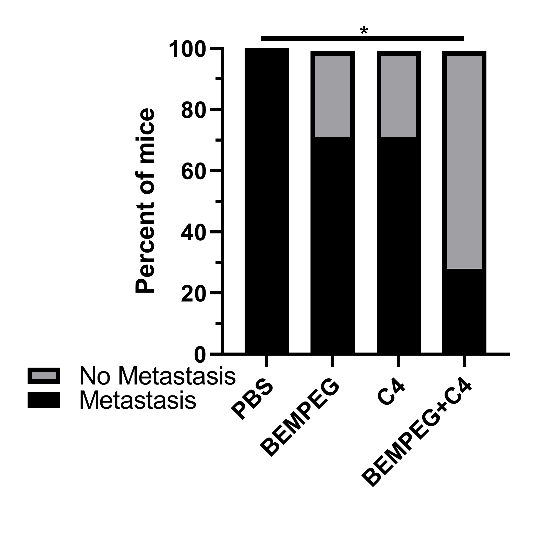  **B** | 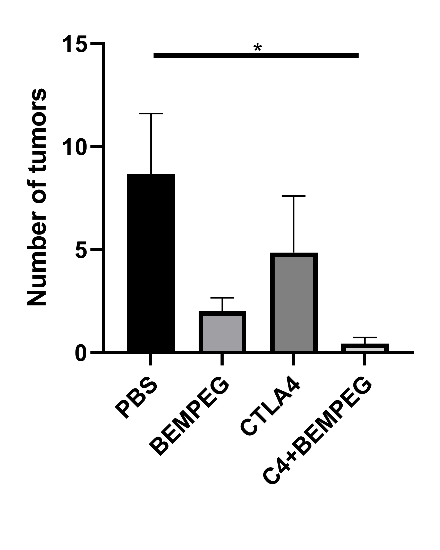  **C** |
| --- | --- | --- |

Supplemental Figure 3. Combined treatments of BEMPEG and anti-CTLA-4 (C4) better prevent metastatic disease compared to monotherapies. A) Tumor volume growth curves are shown, mean±SEM, n≥6. B,C) At day 30, mice were euthanized lungs were harvested and India ink stained as shown in Fig. 2H, and lung metastatic tumor burden was quantified. Fisher’s exact test and a post-hoc pairwise comparison were used for statistical analyses. * = P < 0.05.


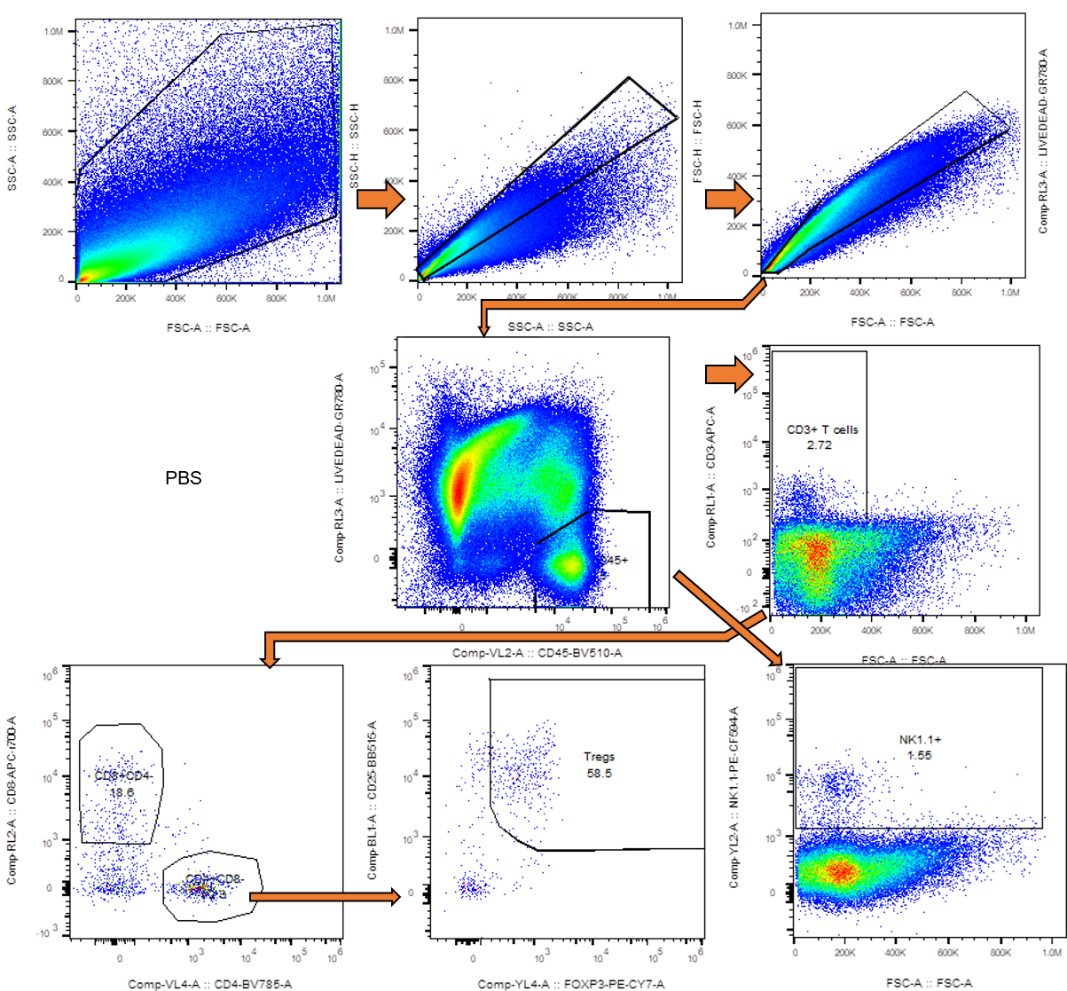

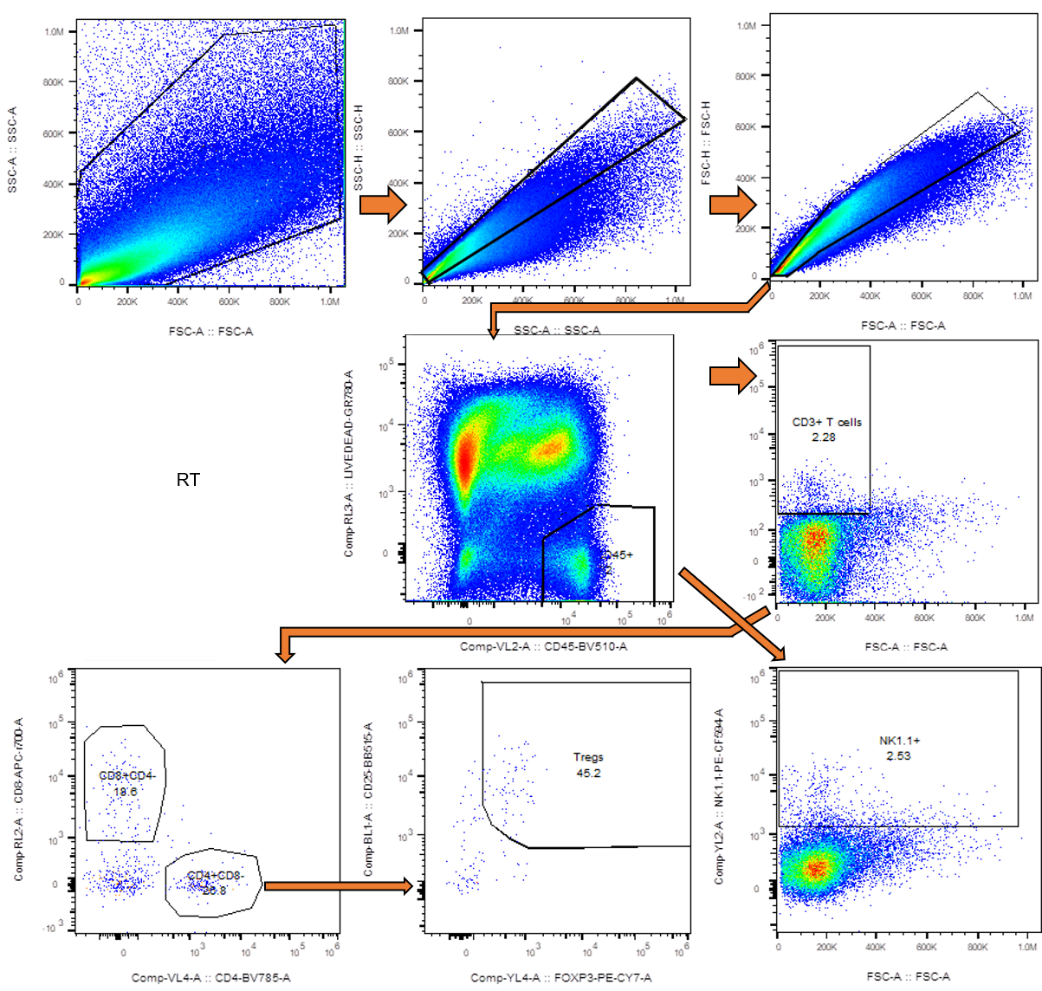

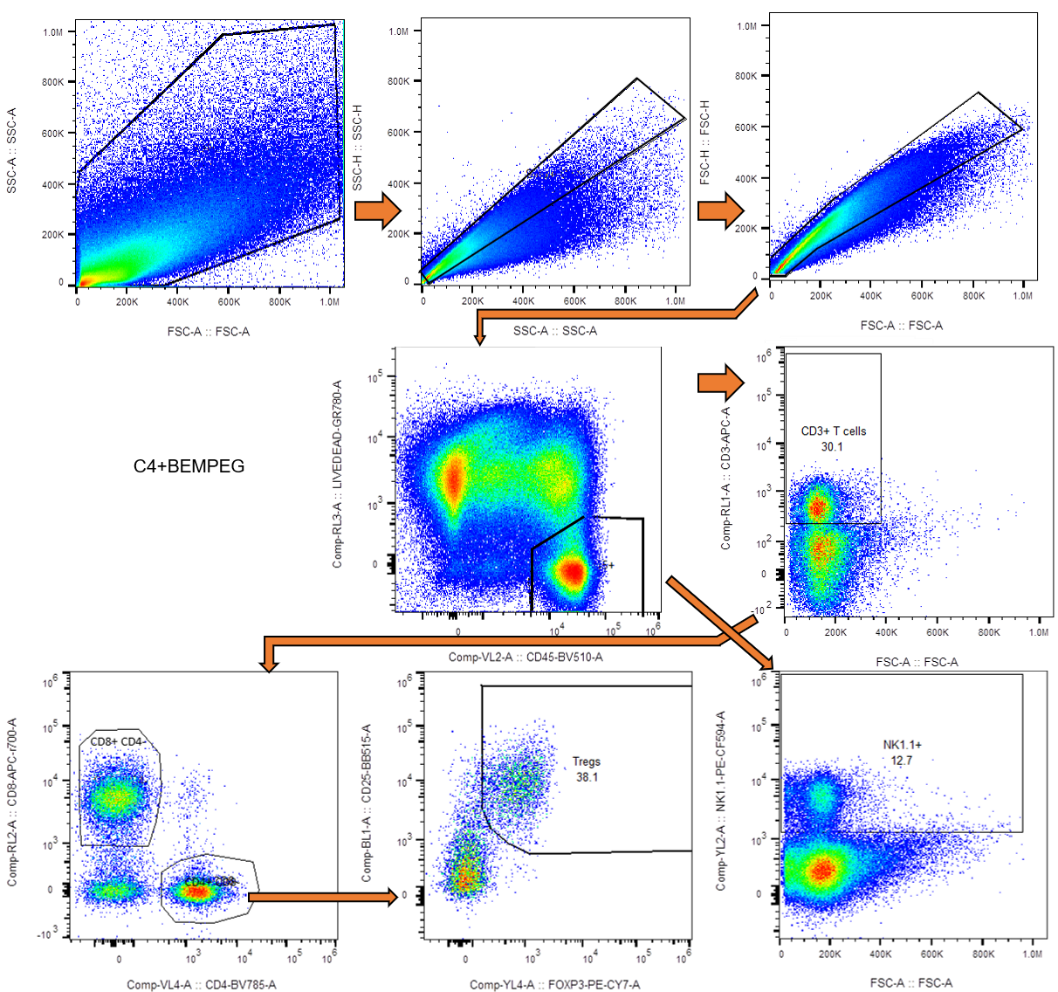

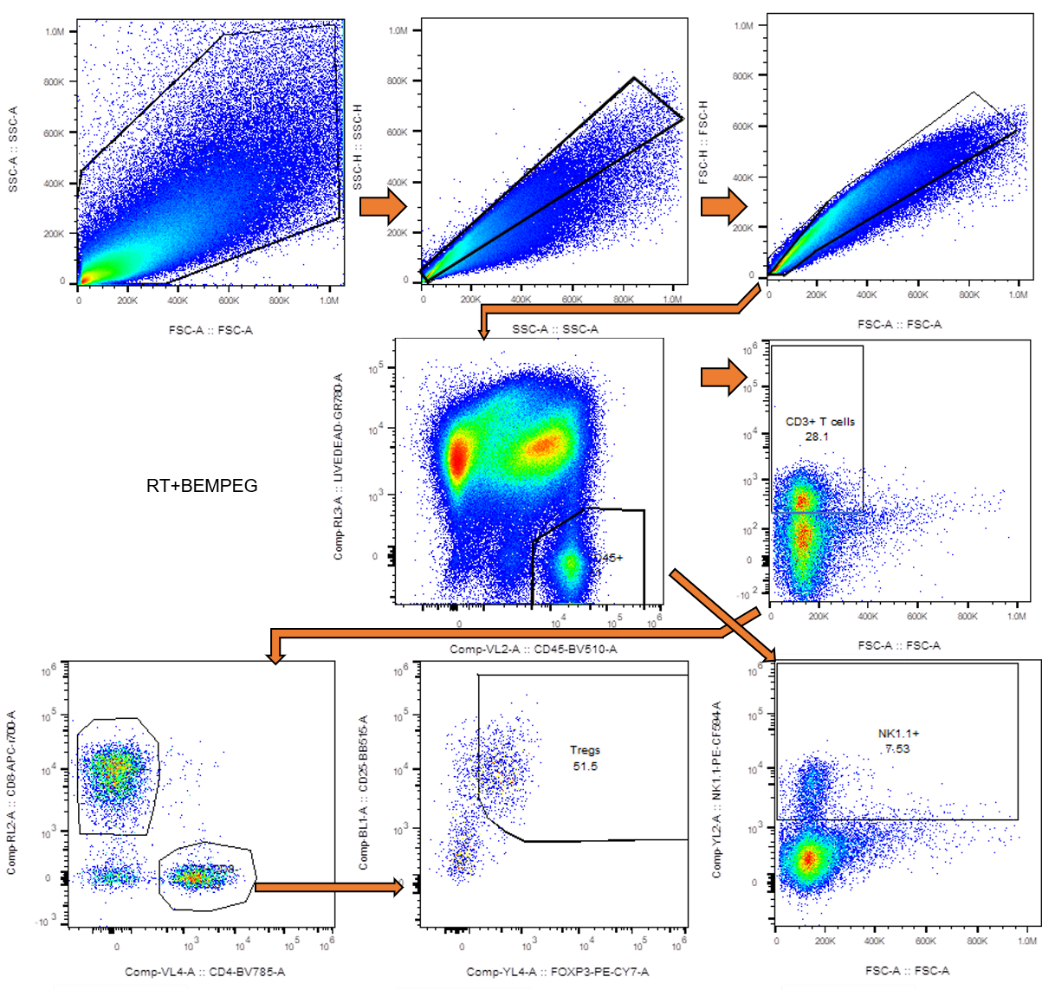

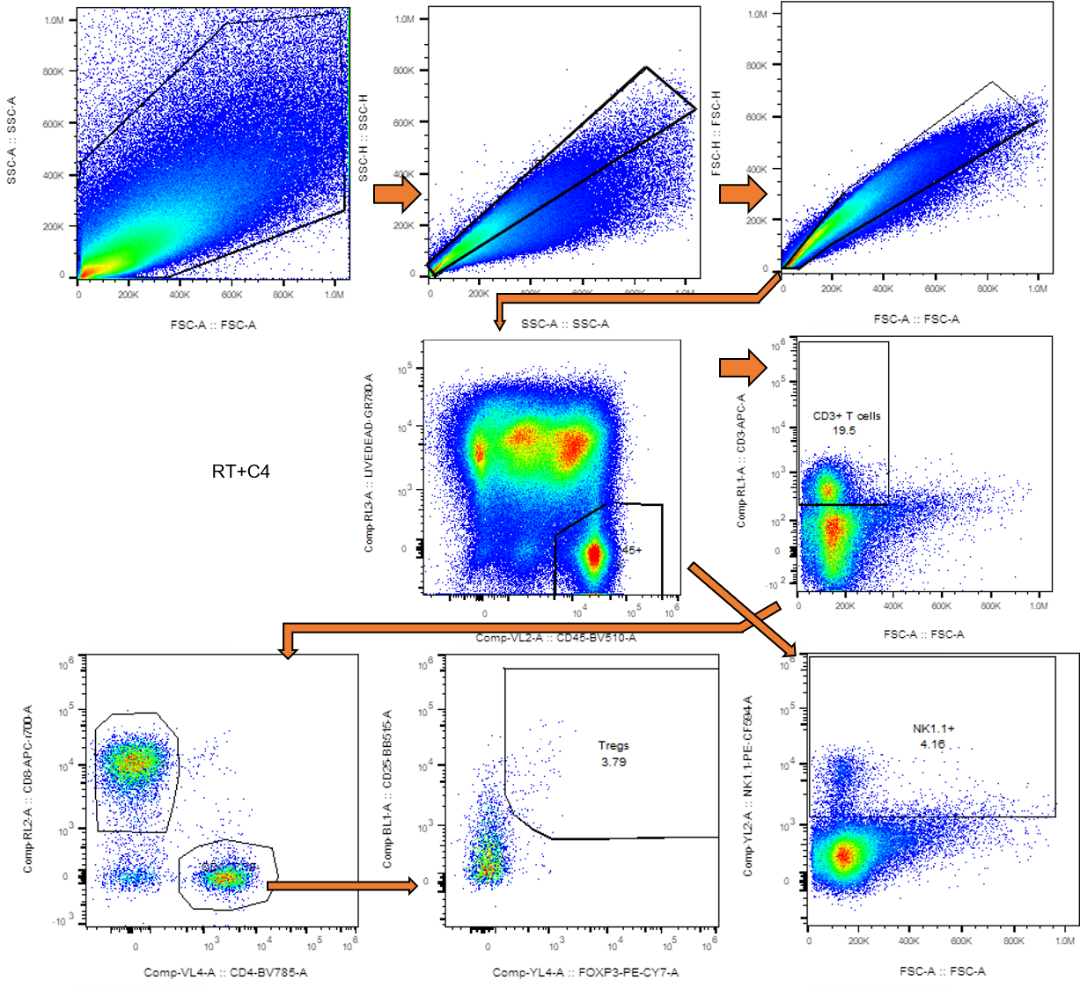

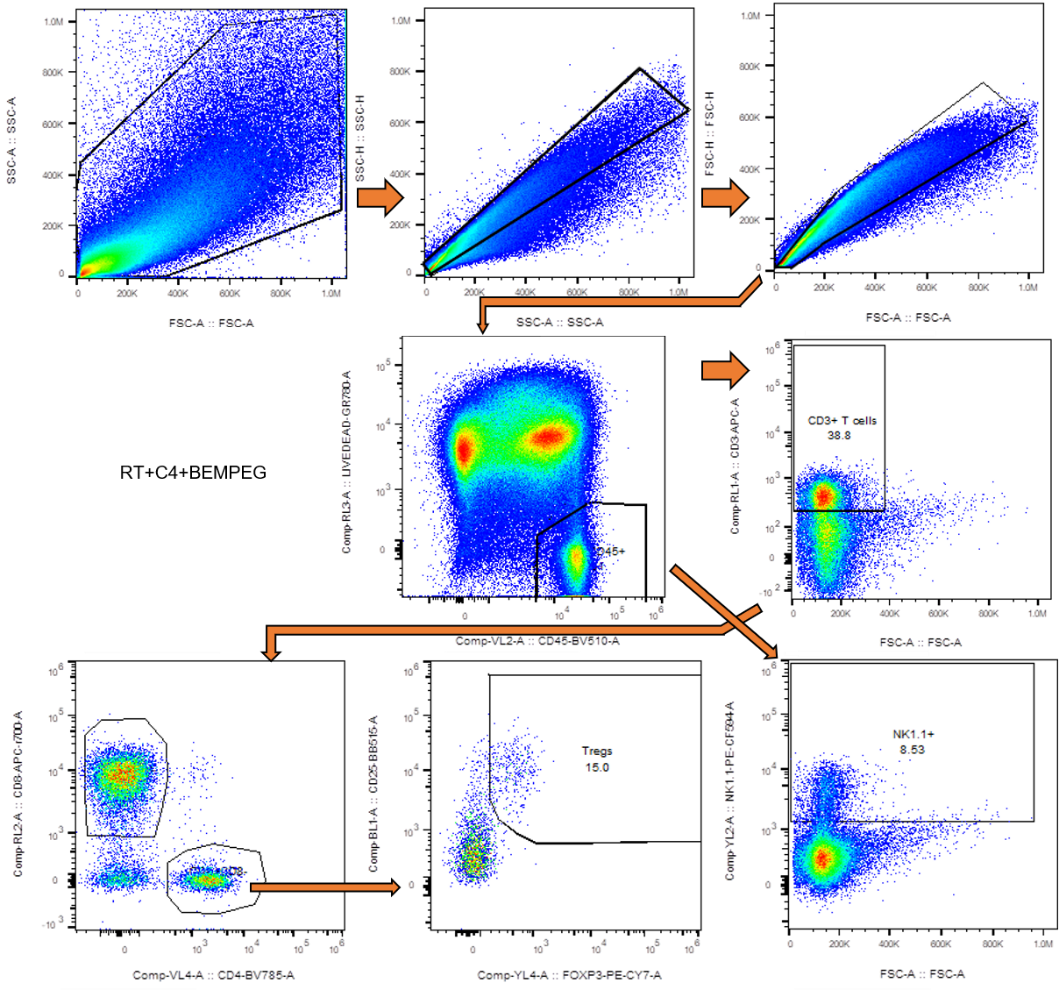


Supplemental Figure 4. The flow cytometry gating strategy used to stain cells in disaggregated tumors is shown. Representative dot plots for each treatment group are shown.

| 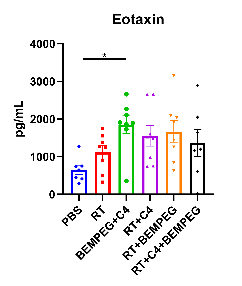 | 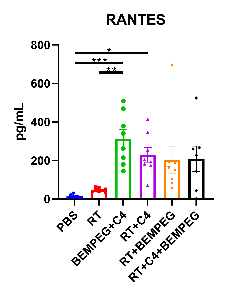 | 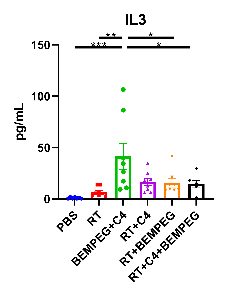 | 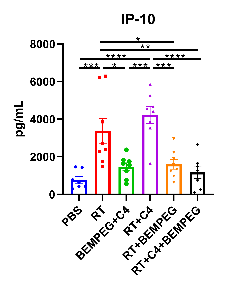 | 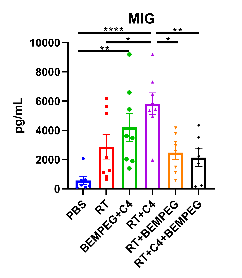 | 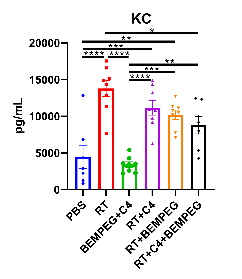 |
| --- | --- | --- | --- | --- | --- |
| 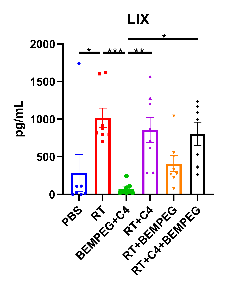 | 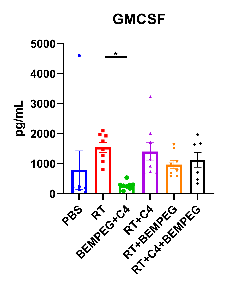 | 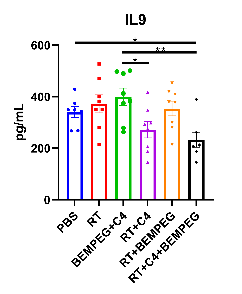 | 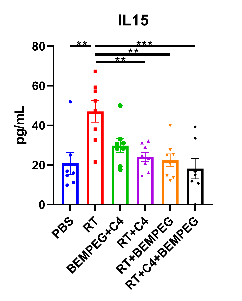 | 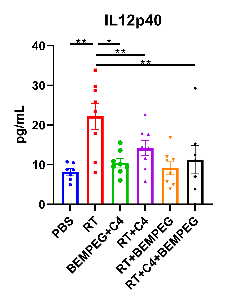 | 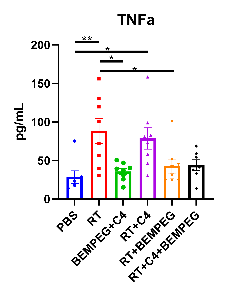 |
| 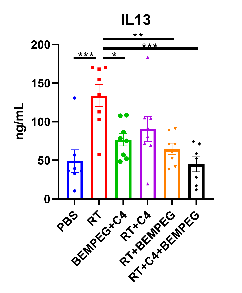 | 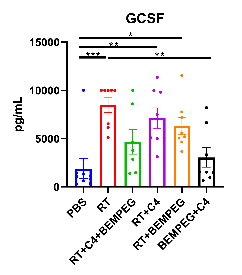 | 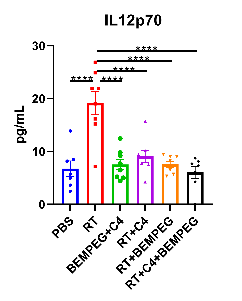 | 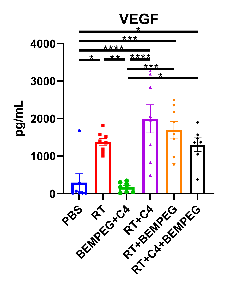 | 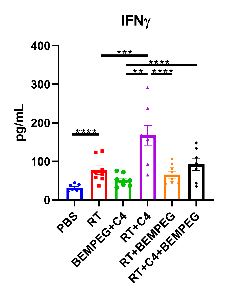 | 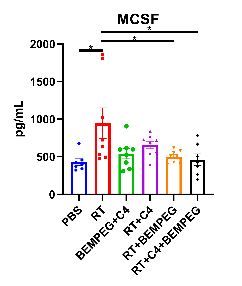 |

Supplemental Figure 5. Multiplex immunoassay analysis was performed to determine concentrations of 25 cytokines and chemokines as seen here and in Fig. 4. A one-way ANOVA followed by a Tukey multiple comparisons test was used to determine statistical differences among cell populations, (mean±SEM, n≥6, **** = P <0.0001; *** = P < 0.001; ** = P < 0.01; * = P < 0.05).

| 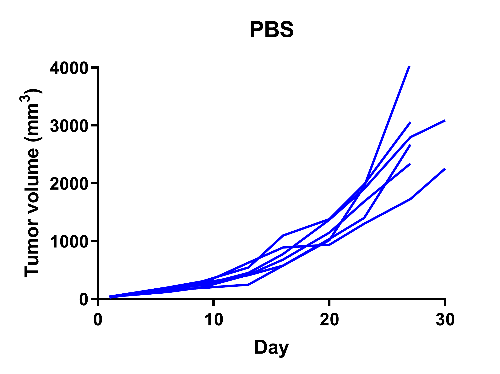  **A** | 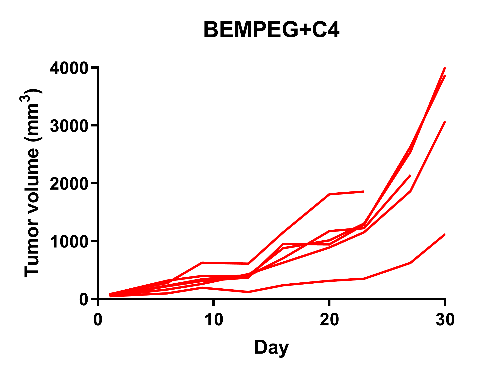 | 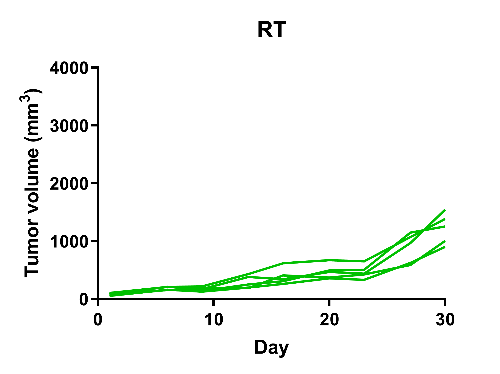 |
| --- | --- | --- |
| 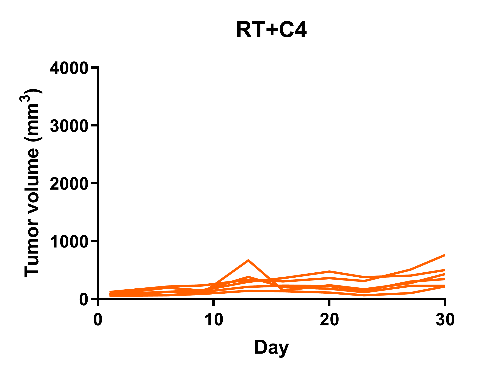 | 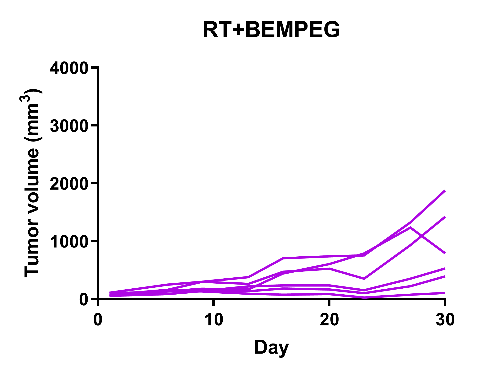 | 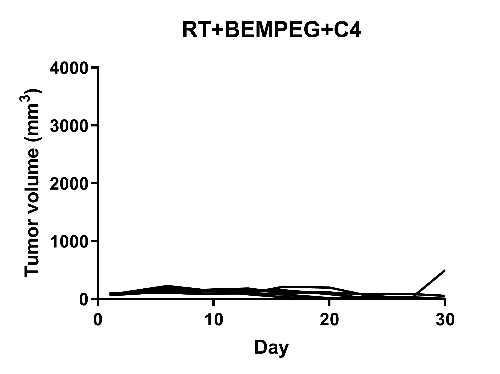 |
| \|  \| BEMPEG+C4 \| PBS \| RT \| RT+C4 \| RT+BEMPEG \| \| --- \| --- \| --- \| --- \| --- \| --- \| \| PBS \| 0.377 \| - \| - \| - \| - \| \| RT \| 0.226 \| <0.001 \| - \| - \| - \| \| RT+C4 \| <0.001 \| <0.001 \| 0.034 \| - \| - \| \| RT+BEMPEG \| <0.001 \| <0.001 \| 0.458 \| 0.810 \| - \| \| RT+BEMPEG+C4 \| <0.001 \| <0.001 \| <0.001 \| <0.001 \| <0.001 \|   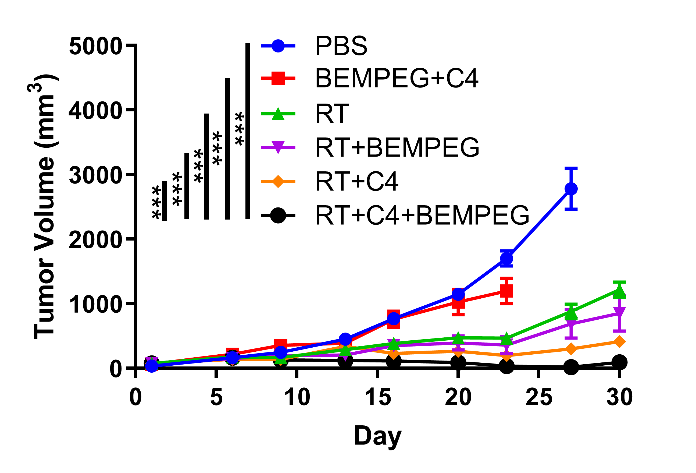  **B** | | |
| 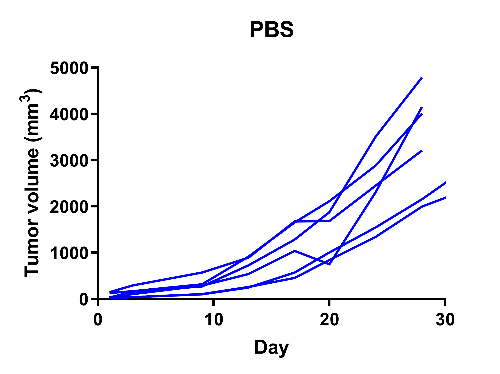  **C** | 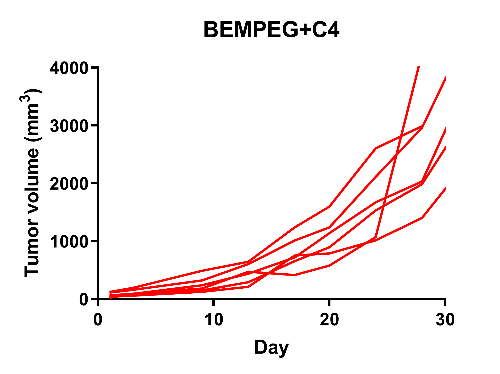 | 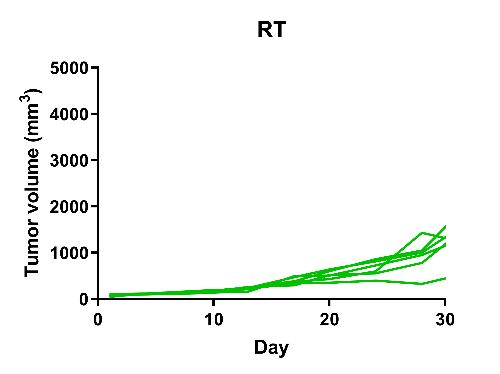 |
| 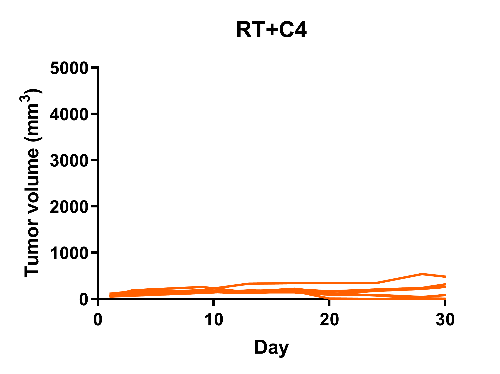 | 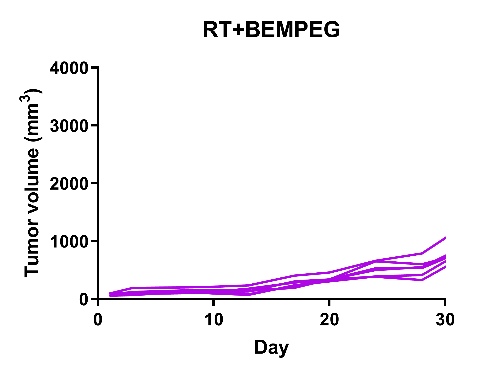 | 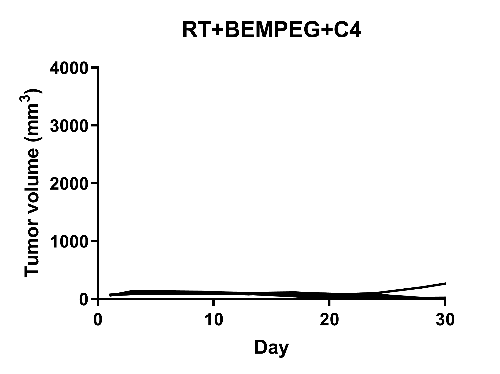 |

Supplemental Figure 6. Tumor volume individual mouse curves (A) and average (B) growth curves are shown comparing RT+C4+BEMPEG to controls (linear mixed effects model, mean±SEM, n≥5, replicate experiment shown in Fig. 1C). C) The individual mouse tumor curves are shown for the experiment shown in Fig. 1C.

| 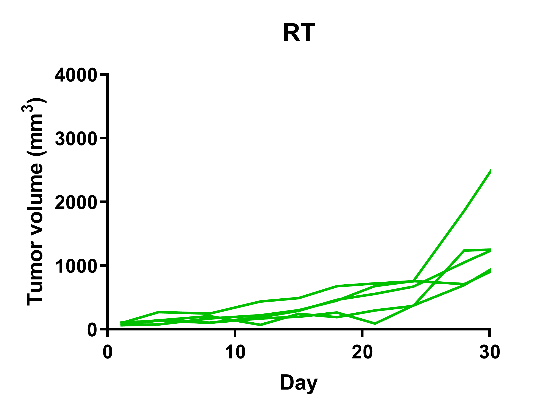  **A** | 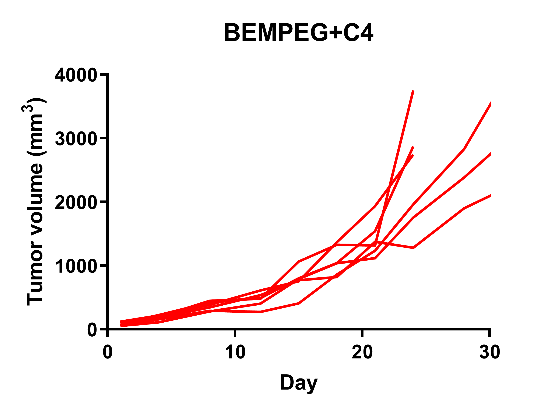 | 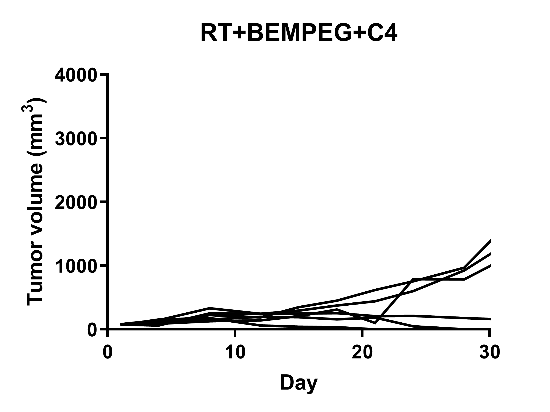 |
| --- | --- | --- |
| 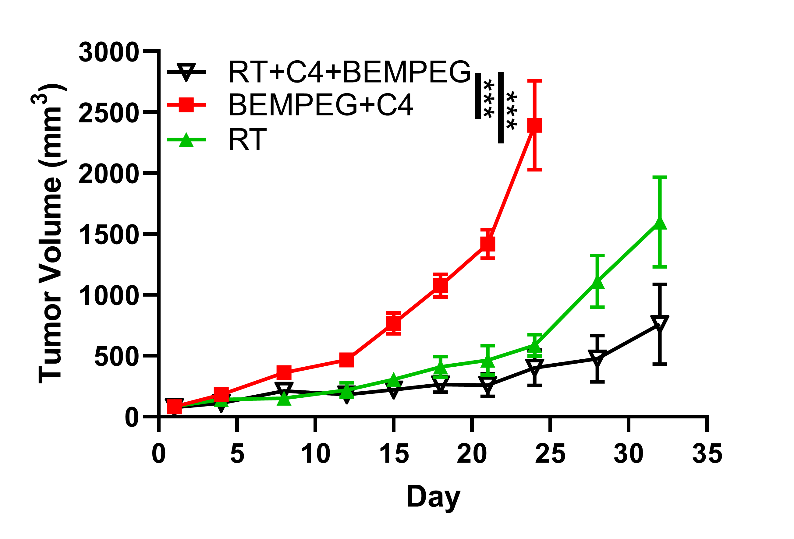  **B** | | |
| 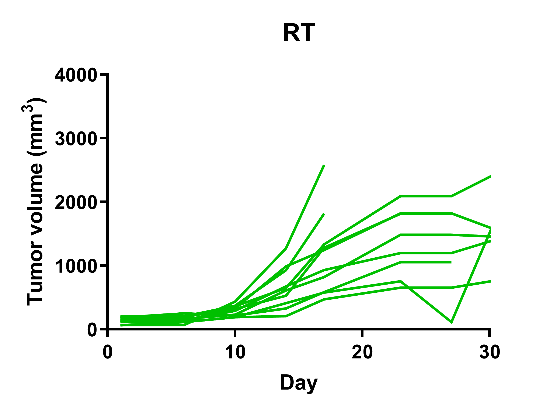  **C** | 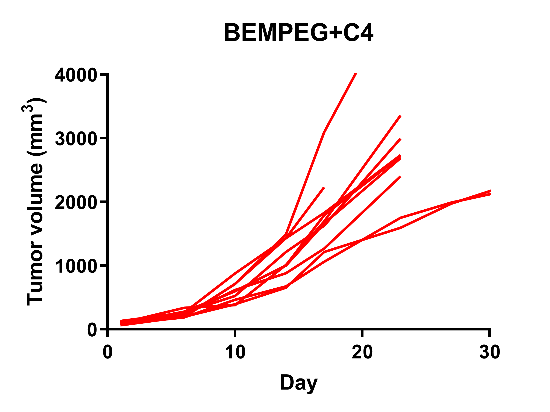 | 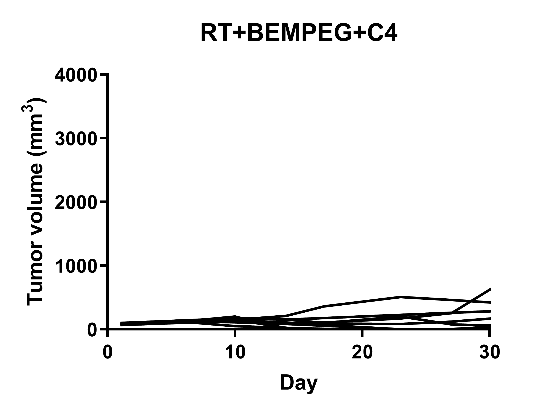 |

Supplemental Figure 7. Tumor volume individual mouse curves (A) and average (B) growth curves are shown comparing RT+C4+BEMPEG to controls (linear mixed effects model, mean±SEM, n=10, replicate experiment shown in Fig. 2C). C) The individual mouse tumor curves are shown for the experiment shown in Fig. 2C.

| 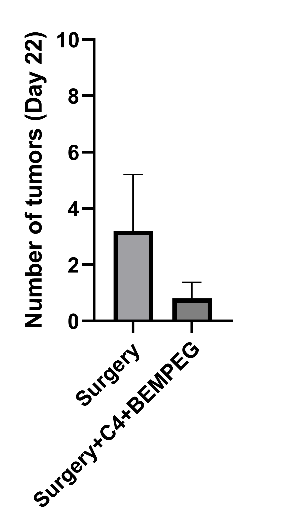  **A** | 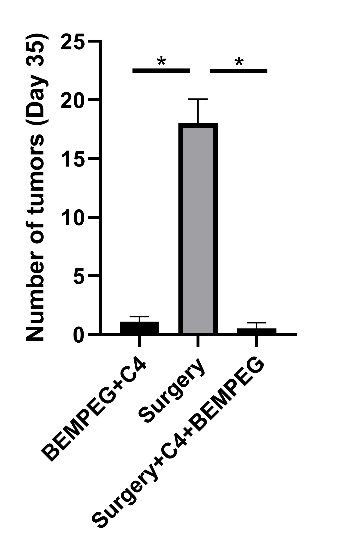  **B** | 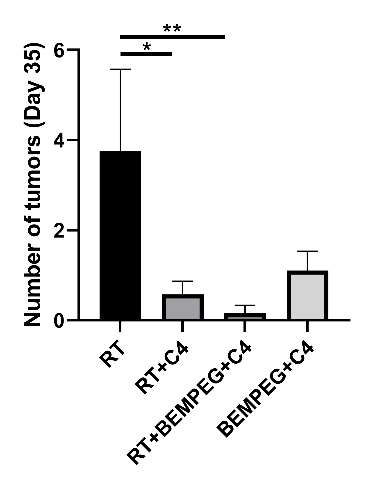  **C** |
| --- | --- | --- |

Supplemental Figure 8. At day 22 (A) and day 35 (B, C) mice were euthanized lungs were harvested and India ink stained as shown in D, and lung metastatic tumor burden was quantified. A) Replicate experiment shown in Fig. 2F. B) Only one repeat was performed as most mice did not survive to day 35. C) Replicate experiment shown in Fig. 2F.
